# MeCP2-driven chromatin organization controls nuclear stiffness

**DOI:** 10.1038/s42003-025-09328-6

**Published:** 2025-12-08

**Authors:** Hector Romero, Anahid Amiri, Maruthi K. Pabba, Hui Zhang, Veronika Berg, Maria Arroyo, Paulina Prorok, Andreas Zhadan, Marah Mahmoud, Nina Trautwein, Bodo Laube, Christian Dietz, Robert W. Stark, M. Cristina Cardoso

**Affiliations:** 1 Cell Biology and Epigenetics, Department of Biology, Technical University of Darmstadt, Darmstadt, Germany; 2 Institute of Materials Science, Technical University of Darmstadt, Darmstadt, Germany; 3 Neurophysiology and Neurosensory Systems, Department of Biology, Technical University of Darmstadt, Darmstadt, Germany

**Keywords:** Nuclear organization, Biophysics

## Abstract

Cellular differentiation is driven by epigenetic modifiers and readers, including the methyl CpG binding protein 2 (MeCP2), whose level and mutations cause the neurological disorder Rett syndrome. During differentiation, most of the genome gets densely packed into heterochromatin, whose function has been simplistically viewed as gene silencing. However, gene expression changes reported in mutations leading to Rett syndrome have failed to be a predictor of disease severity. Here we show that MeCP2 increases nuclear stiffness in a concentration-dependent manner and dependent on its ability to cluster heterochromatin during differentiation. MeCP2-dependent stiffness increase could not be explained by changes in the expression of mechanobiology-related genes, but we found that it is disrupted by Rett syndrome mutations and correlated with disease severity. Our results highlight the impact of chromatin organization on the mechanical properties of the cell as an alternative or complementary mechanism to changes in cytoskeleton components.

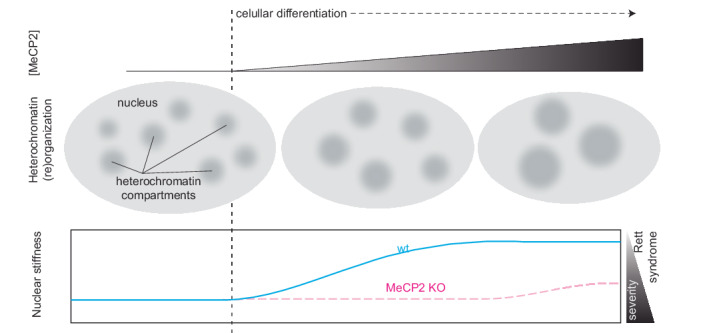

## Abstract (web summary)

A correlative AFM–fluorescence approach elucidates how epigenetic control of chromatin organization contributes to nuclear stiffness and its dysregulation in Rett syndrome.

## Introduction

The (micro)environment surrounding cells is fundamental to understanding the function of these cells. In recent years, the importance of the physical properties of the environment, which drive different biological processes, including proliferation^1^, mobility^2^, and differentiation^3–8^, is gaining special relevance. Changes in the physical properties of the tissues have also been related to the effects of aging^9^ and disease^9,10^.

Physically mediated processes are very complex to study in biological samples. Not only are there differences in the stiffness between tissues^11^, but also among regions in the same tissue, such as different areas of the brain^12^. Indeed, different types of cells located in the same environment respond differently to the same stimulus, which suggests the presence of mechanisms that regulate responses to physical signals. Most studies try to answer this question through the investigation of the changes in cytoskeleton and/or membrane components^11–18^. Interestingly, although the nucleus has been considered to play a major role in mechanical responses^2,17,19–22^, the contribution of the chromatin organization to the differential responses to mechanical stress has been rarely addressed.

Mostly, the nuclear stiffness has been characterized to be dependent on the laminA concentration^11^ and its contribution related to the linker of nucleoskeleton and cytoskeleton (LINC) complex^23^. However, studies showed that substrate stiffness^24–26^, topographical cues^27^, and cellular geometry^28–30^, modulate nuclear organization, chromatin remodeling, and gene expression. These findings indicate that mechanical cues are diverse in nature and selectively regulate the epigenome, with significant implications for physiology and therapeutic applications.

Each human cell contains meters of DNA packed into an ovoid nucleus with a 5–20 µm diameter^20^. To make such a dense packing possible, DNA is condensed into chromatin. However, the condensation of the chromatin is not homogeneous, leading to membraneless compartments within the nucleus: euchromatin (more open and accessible chromatin) and heterochromatin (condensed chromatin). These compartments are highly dynamic and undergo significant reorganization during cell differentiation^31–34^. This reorganization is directed by epigenetic marks and driven by epigenetic readers. One prominent reader is MeCP2, whose levels are dependent on cell differentiation^33,34^. MeCP2 induces the clustering of heterochromatin in a concentration-dependent manner^35^, and changes in its levels or mutations in the MeCP2 gene are linked to diseases, with special relevance to the neurological disorder Rett syndrome (OMIM: #312,750)^36–38^, as MeCP2 is especially abundant in neurons^39,40^.

Taking together the importance of the nucleus in the cell response to mechanical stress and the possibility of changing the organization of the chromatin within the nucleus, we hypothesize that nuclear stiffness is regulated not only by the cytoskeleton but also through dynamic chromatin organization, being dysregulated in disease. In this study, we investigated the effect of this epigenetic reader and chromatin organizer (MeCP2) on nuclear stiffness using atomic force microscopy (AFM) to measure the elastic modulus of nuclei and cells, alongside analyzing chromatin organization. We aimed to determine how MeCP2 level and chromatin clustering influence nuclear stiffness, particularly in the context of cellular differentiation and disease models, including Rett syndrome, and to assess the role of mechanotransduction pathways in these processes. Our results suggest that MeCP2 is a major factor increasing nuclear stiffness in neuronal differentiation systems, and that this function is relevant for the severity of Rett syndrome derived from MeCP2 mutations.

## Results

### The stiffness of the nucleus is independent of the cytosolic cytoskeletal components

**Fig. 1:**
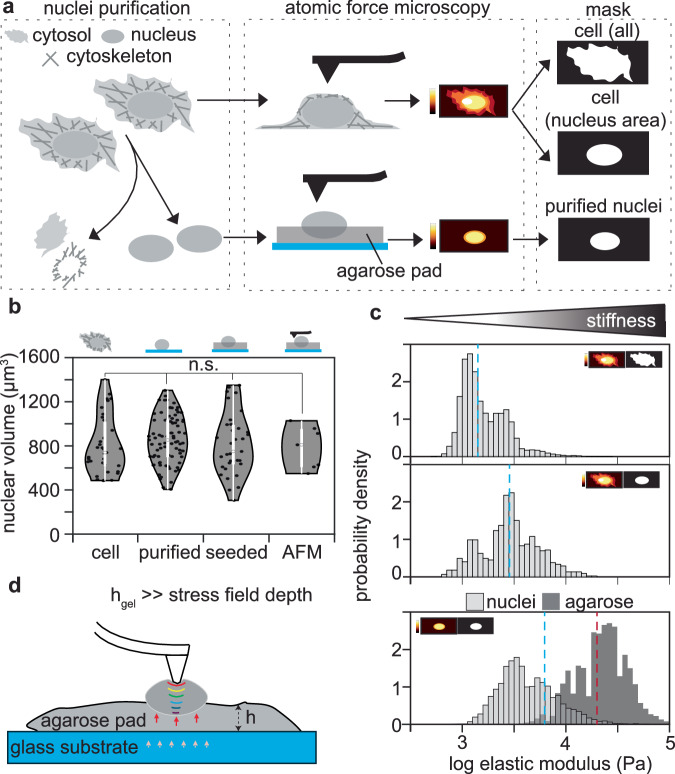
Nuclear stiffness does not depend on the cytosolic components. **a** Scheme of the experiment. C2C12 myoblast nuclei were extracted to remove the cytosolic cytoskeleton. Atomic force microscopy force maps were acquired for the cells and the purified nuclei. The purified nuclei were seeded on top of a 0.5% agarose gel pad. To analyze the data, a mask was used to depict the cell, the nucleus area of the cell, or the purified nucleus. **b** Quantification of the nuclear volume in the different steps of the preparation. 3D confocal analysis of DAPI staining for cells seeded and fixed on glass coverslips (*n* = 35), isolated nuclei deposited on glass coverslips (*n* = 81), or seeded on 0.5% agarose pads (*n* = 35). Individual nuclei (*n* = 7) were selected from the atomic force microscopy images, and volume was calculated to confirm the results obtained in the 3D analysis. To assess the significance, a 2-sided *t*-test was performed to calculate a *p* value. Only significant differences are shown. *: *p* value < 0.05; **: *p* value < 0.001; ***: *p* value < 0.0001. **c** Histogram of elastic modulus values for cells grown on plates, showing measurements from the entire cell area (upper panel, n = 3) and from the nuclear region only (middle panel, *n* = 3). The lower panel (light gray histogram) shows data from isolated nuclei seeded on agarose (*n* = 37), with the agarose stiffness distribution shown in dark gray for comparison (*n* = 4). The median values for each condition are represented in dashed colored lines (blue: cell, nuclear area of the cell and purified nuclei, dark red: agarose) **d** Scheme of the probe–nucleus–agarose interface during a force spectroscopy measurement. Color gradients (red → yellow → green → blue) qualitatively represent the stress distribution across the interface, from high to low. Due to the forces (arrows) mainly from the agarose substrate, we performed a bottom effect cone correction in the elastic modulus calculation. Adapted from ref. ^41^.

We first purified the nuclei to remove all cytosol components, including the major cytoskeleton components (Fig. 1a). Since AFM indentation involves direct physical interactions between the tip and the sample, isolated nuclei exhibited drift during image acquisition. To stabilize them, nuclei were seeded on top of a pre-polymerized agarose pad (>100 µm thick), which constrained lateral motion without exerting compressive forces. The agarose was crosslinked prior to seeding, ensuring that nuclei rested on the surface rather than being embedded within the gel. This setup enabled accurate and reproducible measurements of nuclear elastic modulus.

All measurements in this study were conducted under identical conditions, consistent sample preparation, experimental parameters (tip speed, setpoint, resolution), and methodology, ensuring comparability across samples. Nuclear purification and seeding onto agarose pads were performed in the same physiological buffer used throughout the purification process. Quantification of nuclear volume before and after seeding revealed no significant volume change compared with in situ nuclei (Fig. 1b), confirming the absence of compression or osmotic effects.

The agarose substrate was approximately one order of magnitude stiffer than the nuclei (Fig. 1c). To minimize substrate contributions, indentation data were corrected using the Bottom Effect Cone Correction (BECC) model, which compensates for finite-thickness effects of the supporting layer^41^. For a conical indenter, the contact radius is defined as *a* = δtanθ, and the stress field extends to ~5*a*^42^. Modulus calculations were therefore restricted to a maximum indentation depth of 1 µm, where the stress field (~3.5 µm) remained well within the agarose pad, rendering the underlying glass substrate mechanically irrelevant (see schematic in Fig. 1d). This approach allowed us to extract the intrinsic mechanical properties of the nuclei.

We compared the elastic modulus measurements obtained from whole cells and specifically in the nuclear region to those measured in purified nuclei isolated from the same cell type (Fig. 1c and Table 1). Because the elastic modulus distributions were skewed and contained extreme outliers, we reported median values as a more appropriate measure of central tendency throughout the manuscript. Our results showed that: (i) the nuclear region within the intact cells exhibited higher elastic modulus values, with a median value of 2.9 kPa, compared to that of the whole cell, with a median value of 1.4 kPa; and (ii) the purified nuclei displayed an elastic modulus distribution comparable with that of nucleus area of the cell (median value of 6.1 kPa). The similarity in stiffness distributions between purified nuclei and the nuclear region of intact cells indicates that neither the agarose support nor the purification process introduced compressive or dehydration artifacts^22,43^. In the absence of cytosolic cytoskeletal components, purified nuclei exhibited a more homogeneous modulus distribution, demonstrating that nuclear stiffness is intrinsic and independent of cytoskeletal components, while cytoskeletal coupling contributes primarily to heterogeneity in stiffness.

**Table 1:** Summary of nuclear elastic modulus values across cell types and MeCP2 perturbations

Cell line	Sample	n	Mean elastic modulus (kPa) ± STDV	Median elastic modulus (kPa) ± MAD
C2C12	mb cell	3	1.9 ± 1.3	1.4 ± 0.4
C2C12	mb cell (nucleus area)	3	3.5 ± 2.4	2.9 ± 1.2
-	Agarose	4	22.8 ± 21.1	20.6 ± 9.9
C2C12	mb nuclei	37	11.8 ± 113.0	6.1 ± 2.4
C2C12	mb MeCP2+ low nuclei	48	17.0 ± 79.2	9.5 ± 4.9
C2C12	mb MeCP2+ high nuclei	20	22.5 ± 81.5	9.8 ± 5.3
J1 wt	ESC nuclei	38	16.1 ± 46.2	4.1 ± 2.6
J1 wt	-LIF day 7 (D7) nuclei	44	9.3 ± 98.6	2.3 ± 1.2
J1 wt	-LIF day 14 (D14) nuclei	34	99.2 ± 317.5	20.5 ± 18.2
J1 wt	-LIF day 21 (D21) nuclei	48	29.3 ± 48.8	15.7 ± 8.6
J1 MeCP2 KO	ESC nuclei	37	8.8 ± 56.1	3.2 ± 1.2
J1 MeCP2 KO	-LIF day 7 (D7) nuclei	47	6.1 ± 104.3	1.6 ± 0.5
J1 MeCP2 KO	-LIF day 14 (D14) nuclei	40	8.6 ± 46.9	1.4 ± 0.5
J1 MeCP2 KO	-LIF day 21 (D21) nuclei	37	14.8 ± 56.0	4.2 ± 2.5
J1 NSC wt	NSC nuclei	18	208.2 ± 554.8	30.0 ± 18.0
J1 NSC wt	Neuron nuclei	40	304.3 ± 557.6	113.9 ± 93.9
J1 NSC MeCP2 KO	NSC nuclei	29	51.3 ± 291.7	10.7 ± 4.8
J1 NSC MeCP2 KO	Neuron nuclei	34	54.8 ± 166.6	24.7 ± 12.9
C2C12	Untransfected (mb) nuclei	47	18.8 ± 105.5	2.0 ± 0.5
C2C12	+ pMeCP2 wt nuclei	54	79.6 ± 938.7	6.7 ± 3.9
C2C12	+ pMeCP2 P101H nuclei	22	4.3 ± 4.6	2.9 ± 1.8
C2C12	+ pMeCP2 R106W nuclei	17	2.7 ± 3.3	1.7 ± 0.4
C2C12	+ pMeCP2 R133C nuclei	35	16.4 ± 52.7	6.0 ± 4.3
C2C12	+ pMeCP2 A140V nuclei	29	17.0 ± 31.8	8.3 ± 6.4
C2C12	+pMeCP2 T158M nuclei	32	1.9 ± 2.3	1.4 ± 0.3
C2C12	+pMeCP2 R168X nuclei	16	5.4 ± 37.4	2.5 ± 1.0
C2C12	+pMeCP2 R255X nuclei	22	7.0 ± 23.3	2.5 ± 1.4
C2C12	+pMeCP2 R270X nuclei	29	11.3 ± 11.4	7.2 ± 2.3
C2C12	+pMeCP2 R294X nuclei	34	12.8 ± 31.6	5.8 ± 3.8
J1 NSC MeCP2 KO	NSC nuclei	34	6.3 ± 10.3	4.0 ± 1.3
J1 NSC MeCP2 KO	+pMeCP2 wt nuclei	27	393.0 ± 1305.0	35.4 ± 31.8
J1 NSC MeCP2 KO	+pMeCP2 R106W nuclei	35	19.3 ± 45.1	13.6 ± 6.3
J1 NSC MeCP2 KO	+pMeCP2 A140V nuclei	35	76.0 ± 138.7	22.3 ± 19.1
J1 NSC MeCP2 KO	+pMeCP2 T158M nuclei	41	45.4 ± 520.0	14.3 ± 6.8
J1 NSC MeCP2 KO	+pMeCP2 R168X nuclei	44	40.1 ± 86.4	14.6 ± 9.9

Elastic modulus values are given as mean ± standard deviation (STDV), and median ± median absolute deviation (MAD).

### MeCP2-dependent heterochromatin clustering increases stiffness

**Fig. 2:**
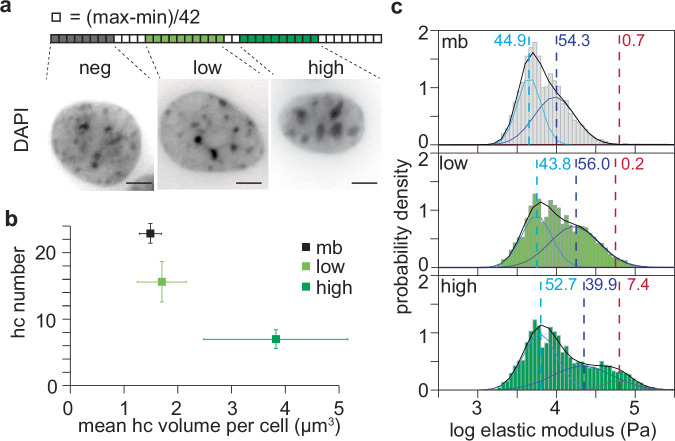
Heterochromatin clustering driven by MeCP2 increases nuclear stiffness. **a** Visualization of the heterochromatin compartments by DAPI staining (high-density foci) in C2C12 myoblasts expressing different levels of MeCP2. The levels were defined by fluorescence-activated cell sorting (FACS), following the gate scheme depicted in the up. Scale: 5 µm. **b** Clustering analysis including the mean and 95% confidence interval of the number of heterochromatin compartments and the average volume of the heterochromatin compartments per cell calculated by 3D confocal microscopy in fixed cells on coverslips and stained with DAPI (*n* cells used: mb = 35; MeCP2 low = 13; MeCP2 high = 10). The violin plot representing the individual measurements is shown in Supplementary Fig. 1. **c** Elastic modulus distribution obtained from several nuclei per fraction, corresponding to different MeCP2 expression levels separated by FACS: mb (n = 35), low MeCP2 fraction (*n* = 48) and high MeCP2 fraction (*n* = 20). A Gaussian mixture model (GMM) with three components was used to highlight differences between conditions. The black line represents the overall model fit; cyan, blue, and red lines indicate the individual subpopulations. Dashed lines mark the mean of each population, and the colored numbers denote the relative weight of each subpopulation (in %), corresponding to the area under the curve.

Having established and validated AFM measurements on purified nuclei, we next investigated the impact of heterochromatin on nuclear stiffness. For this, we took advantage of a method we developed that allowed us to identify cell fractions with distinct, quantified levels of MeCP2^44^. This was accomplished through fluorescent-activated cell sorting (FACS) of myoblasts transfected with a plasmid encoding GFP-tagged MeCP2. The rationale for using myoblasts was that, being adult stem cells, the endogenous level of MeCP2 in these cells is low to undetectable. Ectopic expression of MeCP2 reorganized heterochromatin in a concentration-dependent manner by clustering heterochromatin compartments (Fig. 2a). This reorganization reduced their number while increasing their size (Fig. 2b and Supplementary Fig. 1b, c), consistent with previous reports in these cells^33,35^.

Nanomechanical characterization of the nuclei using AFM revealed an increase in the nuclear stiffness that correlated with the higher MeCP2 concentration (Fig. 2c and Table 1). The median elastic modulus increased from 6.1 kPa in untransfected myoblasts (containing ~0.4 µM MeCP2^44^) to 9.5 kPa in the low MeCP2 level fraction (~11.8 µM MeCP2^44^) and 9.8 kPa in the high MeCP2 level fraction (~131.2 µM MeCP2^44^). Gaussian mixture modeling (GMM) further revealed that (i) low MeCP2 concentrations stiffened the middle population, shifting its modulus from 9.4 kPa to 17.1 kPa, and (ii) at high MeCP2 levels, the proportion of the stiffest population increased markedly, from <1% in untransfected and low-MeCP2 cells to 7.4%. To assess whether MeCP2 overexpression affected the nucleoskeleton, we analyzed Lamin B and Lamin A/C expression. Lamin B levels and distribution remained unchanged (Supplementary Fig. 1d, f, h), while Lamin A/C showed only a minor decrease (Supplementary Fig. 1e, g, h). The (minor) decrease in Lamin A/C with MeCP2 expression would not account for the increased stiffness we measured, but rather, according to the literature, lead to a decrease in nuclear stiffness^11^. Thus, nucleoskeletal organization likely did not underlie the observed increase in stiffness.

Since nuclear volume decreased progressively from myoblasts to low and high MeCP2 conditions (Supplementary Fig. 1a), we examined whether this parameter contributed to the observed changes in stiffness. For each condition (myoblasts, low MeCP2, and high MeCP2), 10 nuclei were individually segmented and masked to determine nuclear volume and the spatial distribution of stiffness within each nucleus. The BECC maps provided voxel-resolved stiffness measurements, from which the mean stiffness value (Pa) was extracted for each nucleus. A two-tailed Spearman’s rank correlation was performed to assess the relationship between nucleus volume (µm³) and BECC mean (Pa). The analysis revealed a very weak negative correlation that was not statistically significant (*ρ* = –0.137, *p* = 0.468, *n* = 30), indicating no evidence of a monotonic association between nuclear size and stiffness in this dataset.

In parallel with the reduction in nuclear size, the number of heterochromatin compartments decreased sharply from myoblasts to low and high MeCP2 conditions (Supplementary Fig. 1b). Notably, the mean heterochromatin volume per compartment remained unchanged in the low MeCP2 group relative to untransfected cells (n.s.), suggesting that the reduced compartment count reflects clustering or fusion of existing heterochromatin domains rather than increased chromatin compaction (Supplementary Fig. 1c). Despite the absence of per-compartment volume changes, nuclear stiffness increased markedly at low MeCP2, implicating clustering-driven chromatin reorganization, rather than hydrostatic pressurization from global compaction, as the principal determinant of stiffening. At high MeCP2 levels, heterochromatin compartments further enlarged while their number declined, accompanied by a corresponding increase in stiffness, consistent with continued coalescence of heterochromatin domains.

To compare chromatin organization with stiffness across conditions, we defined a Heterochromatin Organization Index (HOI) as the ratio of the mean heterochromatin compartment volume, normalized to nuclear volume, to the mean number of compartments per nucleus. Condition-level HOI values were then compared with the corresponding median nuclear stiffness values. This analysis revealed a monotonic increase in nuclear stiffness with increasing HOI, indicating that conditions with fewer but larger heterochromatin domains exhibited higher stiffness. Conditions with more clustered heterochromatin (higher HOI) displayed higher median stiffness values (*E* = 6.0, 9.5, and 9.8 kPa for HOI = 8 × 10^−5^, 17 × 10^−5^, and 140 × 10^−5^, respectively) (Supplementary Table 6). These results are consistent with the notion that heterochromatin coalescence contributes to nuclear stiffening. Because of the limited number of conditions, the trend was interpreted qualitatively rather than as a statistical correlation.

### MeCP2 plays a major role in increasing the nuclear stiffness during neural differentiation

**Fig. 3:**
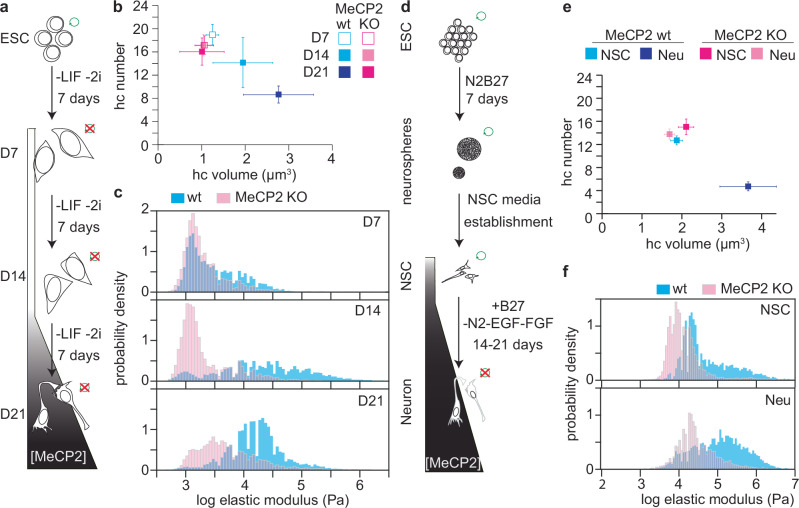
Deletion of MeCP2 severely impairs the increase of stiffness during neuronal differentiation. **a** Differentiation model from embryonic stem cells (ESC) to neurons by leukemia inhibitory factor (LIF) deprivation, with an overview of the renewal abilities (green circle) and the MeCP2 levels after 7, 14, and 21 days (D7, D14, and D21, respectively). **b** Clustering analysis for wt and MeCP2 cells differentiated from ESC by LIF deprivation. 3D confocal analysis was performed in cells fixed and stained with DAPI (n cells used in each condition: wt D7 = 39; wt D14 = 16; wt D21 = 38; MeCP2 KO D7 = 28; MeCP2 KO D14 = 32, MeCP2 KO D21 = 19). The plot represents the mean and 95% confidence interval of each condition. The distribution of the data and the individual values can be found in Supplementary Fig. 3. **c** Comparison of the elastic modulus distribution in wt (blue) and MeCP2 KO (magenta) nuclei from cells differentiated from ESC by LIF deprivation. Nuclei used per condition: wt D7 = 44; wt D14 = 34; wt D21 = 48; MeCP2 KO D7 = 47; MeCP2 KO D14 = 40; MeCP2 KO D21 = 37. A Gaussian mixture model was applied for each dataset for a more quantitative view in Supplementary Fig. 3. **d** Differentiation from ESC to neurons by generating stable neural stem cells (NSCs). **e** Clustering analysis for wt and MeCP2 cells differentiated from stable NSCs. 3D confocal analysis was performed in cells fixed and stained with DAPI (n cells used in each condition: wt NSC = 85; wt neuron = 61; MeCP2 KO NSC = 87; MeCP2 KO neuron = 61). The plot represents the mean and 95% confidence interval of each condition. The distribution of the data and individual points can be found in Supplementary Fig. 3. **f** Comparison of the elastic modulus distributions in wt (blue) and MeCP2 KO (magenta) nuclei from cells differentiated from NSCs. Nuclei used in each condition: wt NSC = 18; wt neuron = 40; MeCP2 KO NSC = 29; MeCP2 KO neuron = 34. A Gaussian mixture model was applied for each dataset for a more quantitative view in Supplementary Fig. 3.

In view of the role of MeCP2 in neurological disease, we investigated whether nuclear stiffness is affected during neuronal differentiation. Therefore, we used a well-characterized model of embryonic stem cells (ESC) differentiation into neurons via LIF deprivation (Fig. 3a). In this model, it is known that: (i) MeCP2 level emerges after 14 days of differentiation^34^; (ii) significant differences in heterochromatin clustering exist between MeCP2 wild type (wt) and MeCP2 knockout (KO) cells^34^; and iii) cellular stiffness increases during differentiation^45^. Therefore, we generated and characterized MeCP2 KO ESC using a MIN-tag strategy (Supplementary Fig. 2a–d). We confirmed the kinetics and absence of MeCP2 in wt and MeCP2 KO cells, respectively (Supplementary Fig. 2e–g), as well as the increase in the neuronal marker NeuN during differentiation (Supplementary Fig. 2h) and the loss of pluripotency by Oct4 immunostaining (Supplementary Fig. 2i, j). Notably, wt and MeCP2 KO cells were indistinguishable based on Oct3/4 and NeuN at each differentiation stage (Supplementary Fig. 2h–k). However, consistent with previous findings^46^, MeCP2 KO nuclei were slightly larger than their wt counterparts (Supplementary Fig. 3a).

We also confirmed impaired heterochromatin clustering in MeCP2 KO cells^34^, characterized by an increased number and reduced size of heterochromatin compartments (Fig. 3b and Supplementary Fig. 3b, c), particularly at differentiation days 14 and 21, when MeCP2 levels peak in wt cells. Using the HOI defined above, we compared heterochromatin organization in wt and MeCP2 KO cells across differentiation stages. In wt nuclei, HOI increased progressively from 5.0 × 10^−5^ at D7 to 10.7 × 10^−5^ at D14 and 37.3 × 10^−5^ at D21, reflecting gradual fusion and growth of heterochromatin domains during differentiation. In contrast, MeCP2 KO nuclei displayed consistently low HOI values (5.9 × 10^−5^, 4.9 × 10^−5^, and 4.5 × 10^−5^ at D7, D14, and D21, respectively), indicating a persistent failure to consolidate heterochromatin (Supplementary Table 6). Note that by D21, wt HOI values are nearly an order of magnitude higher than those in KO nuclei.

We next purified nuclei from each differentiation stage and determined their elastic modulus distributions using AFM. Prior to MeCP2 expression, nuclear stiffness was comparable between wt and MeCP2 KO embryonic stem cells (ESCs), with median values of 4.1 kPa and 3.2 kPa, respectively (Supplementary Fig. 3g and Table 1). After 7 days of differentiation, both groups exhibited reduced stiffness (2.3 kPa in wt and 1.6 kPa in KO). Upon MeCP2 expression at D14, stiffness in wt cells sharply increased (20.5 kPa), driven by the emergence of a stiff subpopulation (peak 64 kPa, 52% of nuclei; Supplementary Fig. 3g), whereas KO cells remained soft (1.4 kPa). After 21 days of differentiation, wt nuclei remained markedly stiffer than their KO counterparts (median 15.7 kPa vs. 4.2 kPa). The modest decrease in wt stiffness and slight increase in MeCP2 KO cell stiffness between D14 and D21 may reflect late-stage chromatin remodeling or compensatory structural adjustments, although further investigation is required to confirm this.

Spearman’s rank correlation (two-tailed) was conducted to assess the relationship between nuclear volume (µm³) and the mean BECC value (Pa). The analysis revealed a very weak, positive correlation that was not statistically significant (*ρ* = 0.144, *p* = 0.273, *n* = 60, 10 individual nuclei from each group). This indicates that there is no evidence of a monotonic association between nuclear volume and stiffness in this dataset.

Both HOI and nuclear stiffness increased in parallel in wt cells, suggesting a direct monotonic relationship between heterochromatin organization and nuclear mechanical reinforcement. In contrast, MeCP2 KO cells maintained low HOI and stiffness, indicating that MeCP2-dependent chromatin organization is required for nuclear stiffening during differentiation. A Spearman’s rank correlation (two-tailed) was used to assess associations between heterochromatin organization (log(HOI)) and the weighted stiffness contribution of soft, mid, and stiff nuclear fractions. The analysis revealed a weak negative trend for the soft fraction (*ρ* = –0.43, *p* = 0.40), a negligible correlation for the mid fraction (*ρ* = 0.26, *p* = 0.62), and a moderate positive trend for the stiff fraction (*ρ* = 0.41, *p* = 0.42). Although none of the correlations reached statistical significance (*n* = 6), the monotonic pattern suggests that increasing heterochromatin clustering is associated with reduced prevalence of soft nuclei and greater stiffening.

To control for variability in differentiation timing at the precursor stage, we established stable neural stem cells (NSCs) from neurospheres derived from ESCs (Fig. 3d and Supplementary Fig. 4a), representing a developmental stage roughly comparable to day 14 of ESC-derived differentiation. MeCP2 KO NSCs exhibited a modest (~16.9%) increase in PAX6 expression (Supplementary Fig. 4b) but maintained equivalent self-renewal capacity, as assessed by 5-ethynyl-2´-deoxyuridine (EdU) incorporation (Supplementary Fig. 4c), and retained their ability to differentiate into neurons (Supplementary Fig. 4d, e), indicating that MeCP2 loss does not impair neural progenitor identity or lineage potential. While NSCs showed detectable MeCP2 expression, overall levels were low compared to those observed in differentiated neurons (Supplementary Fig. 4e, f).

Similar to the LIF deprivation differentiation model, MeCP2 KO NSCs and neurons exhibited larger nuclear sizes than their wt counterparts (Supplementary Fig. 3d). Heterochromatin clustering differences between wt and MeCP2 KO cells became evident after neuronal differentiation. At the NSC stage, both genotypes showed similar heterochromatin compartment number and volume (Fig. 3e and Supplementary Fig. 3e, f), consistent with comparable heterochromatin organization indices (HOI = 100.2 × 10^−5^ in wt and 76.1 × 10^−5^ in KO). Upon differentiation into neurons, wt cells exhibited markedly higher HOI values (534.1 × 10^−5^) relative to KO neurons (73.5 × 10^−5^), reflecting the formation of fewer but larger heterochromatin compartments in the presence of MeCP2 (Fig. 3e and Supplementary Table 6).

AFM measurements revealed that wt nuclei were mechanically stiffer than MeCP2 KO nuclei in both NSCs and neurons (Fig. 3f). Although median stiffness values were comparable between genotypes at the NSC stage (Table 1), the proportion of stiffer nuclei was markedly reduced in MeCP2 KO cells. GMM identified two consistent stiffness populations at approximately 15.9 kPa and 104.5 kPa (Supplementary Fig. 3h). In wt NSCs, the stiffer population accounted for 52.9% of nuclei, whereas it represented only 10.3% in MeCP2 KO NSCs. Upon differentiation, this stiff subpopulation expanded to 80.6% in wt neurons but only 41.2% in MeCP2 KO neurons.

Spearman’s rank correlation (two-tailed) was used to examine the association between heterochromatin organization (log(HOI)) and weighted stiffness fractions across four conditions (NSC wt, Neuron wt, NSC KO, and Neuron KO). The analysis revealed a strong negative monotonic trend between log(HOI) and the mid stiffness fraction (*ρ* = –0.8, *p* = 0.33) and a strong positive trend between log(HOI) and the stiff fraction (*ρ* = 0.8, *p* = 0.33). Although not statistically significant due to the limited number of conditions (*n* = 4), the results suggest that increased heterochromatin clustering is associated with a redistribution from intermediate to stiffer nuclear populations.

Furthermore, to determine whether nucleoskeletal changes could account for the stiffness differences observed in the neuronal system, we analyzed Lamin B and Lamin A/C levels in NSCs. No alterations were detected in either their levels or distribution (Supplementary Fig. 3i–m), ruling out a contribution of the nucleoskeleton to the observed mechanical phenotype.

Collectively, these findings demonstrate that nuclear stiffening accompanies neuronal differentiation and that this process is markedly impaired in the absence of MeCP2, consistent with the reduced heterochromatin clustering observed in MeCP2 KO neurons.

### MeCP2-dependent increase of nuclear stiffness is compromised in mutations linked to Rett syndrome

**Fig. 4:**
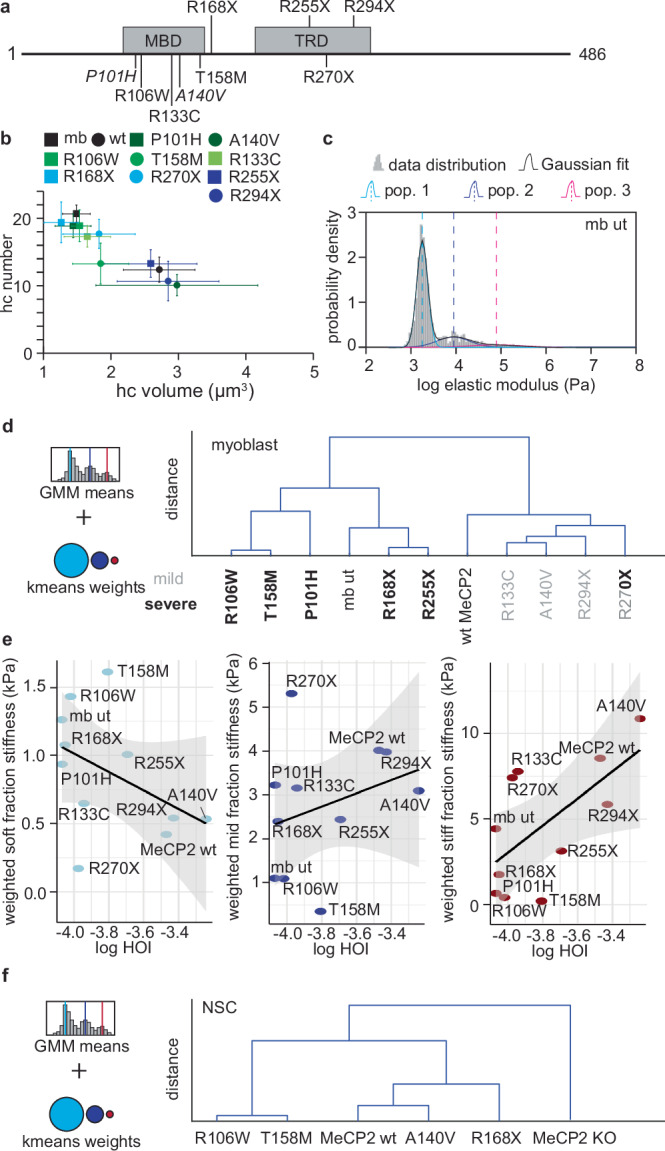
Rett syndrome mutations of MeCP2 impair the increase in nuclear stiffness. **a** Scheme of the main domains of the MeCP2 protein (MBD: methyl binding domain; TRD: transcription repression domain) and the location of the mutations studied. **b** Clustering analysis using 3D confocal microscopy on fixed C2C12 cells, untransfected or transfected with plasmids containing wt or mutant MeCP2 cDNA. Cells were fixed with formaldehyde and stained with DAPI (n number used for each condition: mb = 35, wt MeCP2 = 40; P101H = 15; R106W = 13; R133C = 20; A140V = 14; T158M = 14; R168X = 7; R255X = 20; R270X = 29; R294X = 19). Only transfected cells were analyzed; however, their expression levels were heterogeneous, which is reflected in the variability observed in the atomic force microscopy results. The mean and 95% confidence interval of the number of heterochromatin compartments and the average volume of the compartments per cell are represented. The violin plots containing the individual data are shown in Supplementary Fig. 5b, c. **c** Representative histogram of the elastic modulus with Gaussian mixture model (GMM) fits. Nuclei were purified from untransfected C2C12 cells (mb ut), seeded on an agarose pad, and analyzed by atomic force microscopy to determine their elastic modulus. A three-component GMM was applied, showing the overall model fit (black line) and the individual subpopulations (cyan, blue, and magenta lines). Histograms of the elastic modulus values for wild type MeCP2 and mutant nuclei are provided in Supplementary Fig. 5d. **d** Dendrogram showing the effect of Rett mutations on myoblasts based on elastic modulus values distribution. Elastic modulus histograms were fitted with a GMM as described above to obtain the mean modulus values of each subpopulation (Supplementary Fig. 5g). In addition, in order to make the populations comparable, all data were pulled together and fitted to 3 populations, followed by the assignment of the individual data to the three populations based on k-means to obtain the weight of the populations (see Supplementary Fig. 5f). All population means and weights were normalized using z-scores, and Euclidean distances were calculated and represented in a dendrogram. The mutants were classified into mild (gray) and severe (bold) based on previous publications. R270X is variably classified as mild or severe in the literature, likely due to differences in clinical scoring parameters and diagnostic criteria applied across cohorts. **e** Relationship between heterochromatin organization and stiffness contributions of nuclear subpopulations. Linear regressions showing the relationship between the heterochromatin organization index (HOI, log₁₀-transformed) and the weighted stiffness of the soft (left), mid (center), and stiff (right) nuclear fractions across MeCP2 mutants. Weighted stiffness values were calculated by multiplying the proportion of each population by its representative elastic modulus acquired from k-means clustering (1.7, 7.4, and 40.7 kPa for soft, mid, and stiff, respectively). The analysis revealed a negative trend for the soft fraction (*β* = –0.68, *R*^2^ = 0.20), a weak positive trend for the mid fraction (*β* = 1.50, *R*^2^ = 0.09), and a significant positive relationship for the stiff fraction (*β* = 7.91, *R*^2^ = 0.40, *p* = 0.037), indicating that increased heterochromatin organization (higher HOI) is associated with greater mechanical stiffening of nuclei. **f**. Dendrogram of the Rett mutant rescue of NSC MeCP2 KO based on the elastic modulus distribution. The procedure was done as described for **d**, based on GMM populations (Supplementary Fig. 5g, h) and the k-means weights (Supplementary Fig. 5i).

To determine whether MeCP2’s role in increasing nuclear stiffness is altered in the neurological disorder Rett syndrome, we analyzed mutations and truncations known to cause the disorder, including several of the most frequent variants (R106W, R133C, T158M, R168X, R255X, R270X, and R294X; Fig. 4a)^47–49^. Also, we included the P101H and A140V mutations (Fig. 4a) due to their distinctive heterochromatin-binding and clustering behaviors^50^: P101H binds 5mC similarly to wt MeCP2 but fails to cluster heterochromatin, whereas A140V shows stronger binding than wt and generates larger, irregular heterochromatin clusters. We confirmed previously reported clustering behaviors of the mutants using 3D confocal analysis in C2C12 myoblasts (Fig. 4b and Supplementary Fig. 5b, c)^50^. Quantitative analysis of the heterochromatin organization index classified the mutants into three categories: low (<10 × 10^−5^), intermediate (10–30 × 10^−5^), and high (>30 × 10^−5^). Mutants P101H, R106W, and R168X exhibited HOI values comparable to untransfected myoblasts (8–10 × 10^−5^), consistent with impaired heterochromatin coalescence. In contrast, A140V, R294X, and wt MeCP2 showed substantially higher HOI values (≥30 × 10^−5^), reflecting pronounced domain consolidation. T158M displayed an intermediate HOI (15.6 × 10^−5^), in agreement with its partial clustering phenotype, and grouped with R270X, R133C, and R255X, which also fell within the intermediate range (10–20 × 10^−5^) (Supplementary Table 6).

Using the myoblast cells as a screening model, we transfected cells with plasmids encoding either wt GFP-tagged MeCP2 or mutant GFP-tagged MeCP2 and selected the samples that showed 50–70% transfection efficiency before purifying the nuclei for AFM measurements. This guarantees similar numbers of nuclei expressing MeCP2 wt or mutants thereof among the different conditions to be used in the subsequent AFM measurements.

Given the differences in elastic modulus distributions among mutants (Supplementary Fig. 5d), as well as in the populations means when using a Gaussian mixture model (Fig. 4c), we used k-means analysis to group the data into three consistent populations with elastic moduli of 1.7, 7.4, and 40.7 kPa based on the distribution of all the data collected. This allowed us to distribute the elastic modulus of each mutant into these three categories for a direct comparison (Supplementary Fig. 4f). Because both the means of the populations in the Gaussian mixture model and the percentage of the data in each common category are relevant for the stiffness phenotype, we performed a cluster analysis to combine all the information and group the mutants according to their stiffness ability (Fig. 4d). The mutants clustered into three different clusters: one, composed by R106W, T158M and P101H, showed softer nuclei than the untransfected cells; a second one, composed by R168X and R255X, showed no difference compared to the untransfected cells; and a third one, including the mutants R133C, A140V, R294X and R270X showed an increase in the stiffness and were closer to the stiffness obtained for the wt MeCP2. Interestingly, this stiffness distribution was related to the phenotypes described for patients containing these mutations^36,51–55^, being the milder phenotypes closer to wt (represented by non-bold characters in Fig. 4d) and the severe phenotypes closer to the untransfected myoblasts (represented by bold characters in Fig. 4d). R270X is variably classified as mild or severe in the literature, likely due to differences in clinical scoring parameters and diagnostic criteria applied across cohorts.

For each MeCP2 mutant, linear regression analyses were performed to assess how heterochromatin organization (log₁₀HOI) relates to stiffness contributions from the soft, mid, and stiff nuclear populations (from GMM analysis of AFM data). The analysis revealed a negative trend between HOI and the stiffness of the soft fraction (*β* = –0.68, *R*^2^ = 0.20, *p* = 0.17), indicating a reduction of compliant nuclei as chromatin clustering increased. In contrast, the mid fraction showed a weak, non-significant positive association (*β* = 1.50, *R*^2^ = 0.09, *p* = 0.37). A stronger positive correlation was observed for the stiff fraction (*β* = 7.91, *R*^2^ = 0.40, *p* = 0.037), demonstrating that conditions with higher HOI values, reflecting greater heterochromatin coalescence, contained nuclei with markedly increased stiff subpopulation stiffness. Together, these results support that heterochromatin clustering (higher HOI) is accompanied by a redistribution of nuclei toward stiffer mechanical states, with a concurrent reduction in the contribution of soft nuclei (Fig. 4e).

We next validated the stiffness phenotypes of selected MeCP2 mutants in the neural system by genetic rescue of MeCP2 KO NSCs with representative variants from each stiffness cluster identified in the myoblast model (R106W and T158M for the softest, R168X for the intermediate, and A140V for the stiffest group; Fig. 4f and Supplementary Fig. 5g–i). Consistent with the myoblast data, all MeCP2 mutants increased nuclear stiffness relative to the MeCP2 KO, although none reached the levels of the wt MeCP2 rescue. Notably, the low endogenous MeCP2 level in untransfected myoblasts (~0.4 µM) was sufficient to confer an intermediate stiffness comparable to the R168X mutant. In contrast, in the true MeCP2 KO NSC background, the untransfected cells were the softest, and even the softer mutants (T158M and R106W) produced a detectable stiffening effect (Fig. 4f and Supplementary Fig. 5g–i). The relative stiffening hierarchy was preserved, with T158M and R106W showing the lowest stiffness, R168X displaying intermediate phenotype, and A140V clustering with wt MeCP2 as the stiffest among the mutants. These findings suggest that the stiffness phenotypes associated with MeCP2 mutations are intrinsic and reproducible across the myoblast and neural systems, supporting a role for MeCP2-mediated heterochromatin organization in nuclear mechanical reinforcement.

### Stiffness changes related to MeCP2 are not linked to general changes in mechanobiology gene expression

**Fig. 5:**
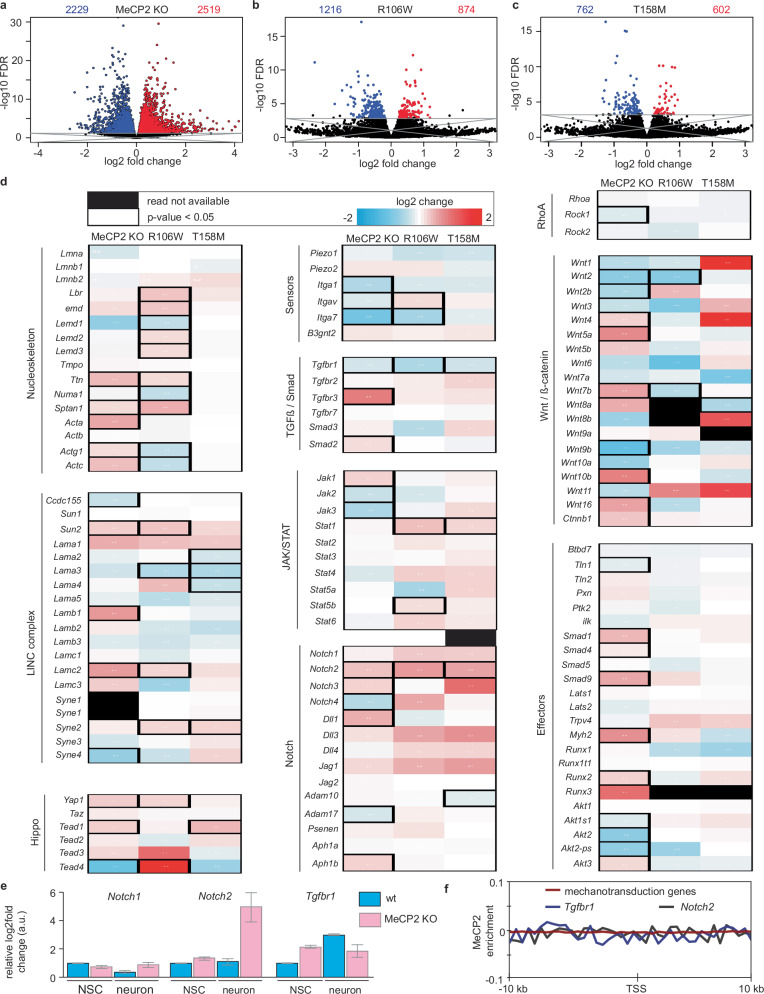
MeCP2 regulates the expression of the mechanotransduction-related genes *Notch2* and *Tgfbr1.* Volcano plots displaying the changes in overall expression of genes by the deletion of MeCP2 (**a**), or the mutation R106W (**b**), or T158M (**c**). Blue and red represent down- and up-regulated genes, respectively, with a false discovery rate (FDR) > 0.05. **d** Gene arrays for the main components of the nucleoskeleton and mechanotransduction pathways and their change due to MeCP2 deletion or mutation. Black outline: significant changes; black boxes: no reads available for these loci in the dataset. **e** Analysis of the expression of relevant genes in qRT-PCR in neural stem cell (NSC) differentiation. The bar plot shows the average and lines the standard deviation of three biological replicates, each of them done with three technical replicates. **f** Chromatin immunoprecipitation analysis of MeCP2 in 20 kb around the transcription start site (TSS) of the genes studied in **d**, as well as specifically for *Tgfbr1* and *Notch2*.

Since chromatin reorganization is a known mechanism for altering gene expression, we explored whether the changes observed in the absence of MeCP2 or due to mutation could stem from alterations in the expression of genes that regulate the mechanical properties of the cell. For this purpose, we analyzed RNA-seq datasets from relevant samples, including brain cortex from MeCP2 KO models, excitatory neurons harboring R106W and T158M. An analysis of global gene expression changes associated with MeCP2 absence or mutation revealed a balance of upregulated and downregulated genes across all datasets (Fig. 5a–c), indicating a dual role of MeCP2 as both a transcriptional activator and repressor.

We next focused on genes related to the nucleoskeleton^56^ and/or implicated in mechanotransduction pathways^15,16,57^, including sensors, receptors, transductors, and effectors (Fig. 5d). Interestingly, among these, only two genes showed significant and consistent changes across all conditions tested: *Tgfbr1* encoding for TFG-ß receptor I (TGFBR1), and *Notch2* encoding for Notch2 (Fig. 5d). These changes were validated using reverse transcription followed by quantitative polymerase chain reaction (RT-qPCR) (Fig. 5e). We further assessed the dynamics of these genes in the differentiation models used. During the differentiation of wt NSCs to neurons, *Notch2* levels remained constant; however, in MeCP2 KO cells, its expression increased more than fourfold (Fig. 5e). In contrast, Tgfbr1 mRNA levels increased during wt differentiation but remained unchanged in MeCP2 KO cells. Notably, at the NSC stage, Tgfbr1 levels in MeCP2 KO cells were already elevated compared to their wt counterpart (Fig. 5e).

To confirm whether the observed changes in *Tgfbr1* and *Notch2* expression were directly driven by MeCP2 binding to their regulatory elements, we analyzed chromatin immunoprecipitation sequencing data of MeCP2 from the mouse brain. The results showed no significant enrichment of MeCP2 binding, neither in the regulatory regions of mechanotransduction genes, nor in the regulatory regions (±10 kb of the transcription start site) of the two genes that were significantly changed (*Tgbfr1* and *Notch2*). These findings indicate that the changes in the expression of *Tgfbr1* and *Notch2* were not due to direct MeCP2 binding but rather arose from the reorganization of the heterochromatin caused by MeCP2 loss or mutation.

## Discussion

In this work, we demonstrated that MeCP2 changes the physical and mechanical properties of the nucleus through its heterochromatin clustering ability. Specifically, an increased MeCP2 concentration correlates with increased nuclear stiffness (Fig. 2 and Table 1). This correlation extends to differentiation systems where MeCP2 levels rise, such as neural differentiation (Fig. 3). Furthermore, we revealed that these changes in the physical properties are disrupted by mutations that lead to Rett syndrome (Fig. 4). Finally, we showed that these alterations were not a consequence of widespread dysregulation of mechanotransduction genes (Fig. 5), but rather the direct result of chromatin reorganization orchestrated by MeCP2.

The nucleus is the largest and stiffest organelle within the cell, playing a critical role in cellular mechanics. Studies have identified several nuclear components crucial to these mechanical functions, including nuclear membrane^58,59^, lamin^23,60^, and nuclear actin^61^. While studies acknowledged chromatin as a contributor to nuclear stiffness^61^, it is often considered in the context of its interaction with lamins^60,62^ or in relation to DNA damage, which facilitates the accessibility of repair factors^63,64^. Here, we demonstrated that MeCP2-driven heterochromatin clustering (Fig. 2b) alone is sufficient to enhance nuclear stiffness (Fig. 2c), consistent with previous reports showing that increased heterochromatin density leads to a stiffer nucleus^65^. We propose that the ability of MeCP2 to interact strongly with heterochromatin, as well as to self-associate, facilitates crosslinking that progressively increases the elastic modulus until either saturation of the binding sites or steric hindrance is reached, thereby limiting further compaction and stiffness.

Studies on the relevance of nuclear stiffness in cellular function have focused on two major points: mechanical stress adaptation and mechanoreception. Mechanical stress adaptation (in particular in muscle) is mostly dependent on the lamin A concentration, as its levels increase with tissue stiffness^11^. Mechanoreception also relies on the mechanical properties of the nucleus for cellular proprioception and mechanotransduction. In proprioception, the physical stimuli applied to cells are only sensed upon nuclear deformation^2^. In mechanotransduction, the import of key transcription factors to the nucleus is controlled by nuclear stiffness. For instance, factors such as YAP are imported into the nucleus only when the mechanical stimuli are recognized by the nucleus^66,67^. In the brain, where lamin A levels are low^11^, we found that chromatin organization (Fig. 3c, f) rather than lamin A (Supplementary Fig. 3k–l) determines nuclear stiffness, rather than being only a response to mechanical stress^19,68,69^. Neurons, which have higher MeCP2 levels than glial cells^70,71^, may utilize this mechanism to elicit distinct responses from glial cells to identical mechanical stimuli.

The temporal dynamics of MeCP2 expression during brain development have been well-documented^72–74^. In our investigation into the role of MeCP2 in nuclear stiffness, we identified two genes, Notch2 and Tgfbr1, that exhibited significant changes in MeCP2 KO mice (Fig. 5e, f). Both factors are implicated in neuronal maturation. The Notch signaling pathway is downregulated during neurogenesis^75^, with Notch2 specifically reported to inhibit neuronal differentiation^76,77^. In contrast, TGFBR1 is active in neurons, and its downregulation reduces survival and maturation of the newborn neurons during neurogenesis^78^. Intriguingly, TGFBR1 is upregulated in other Rett mutations, such as R255X^79^, which may contribute to the previously reported deficits in neurite maturation observed in MeCP2 KO and Rett syndrome^80,81^. Multiple studies have demonstrated interactions between Notch2 and TGF-β signalling^82–84^, primarily in the regulation of cell fate determination and extracellular matrix remodeling^85–88^. Although we cannot exclude a possible role of these proteins in modulating nuclear mechanical properties, there is currently no direct evidence supporting such an effect. Instead, our findings suggest that chromatin organization may play a more prominent role in determining nuclear stiffness.

Our analysis of Rett-associated MeCP2 mutations revealed a correlation between nuclear stiffness and disease severity^36,51–55,89^. Mutations clustering with wt MeCP2 generally aligned with milder phenotypes, while those deviating from the wt cluster correlated with more severe cases (Fig. 4d, f). This would explain why most of the Rett variants of MeCP2 are partially deficient in reorganizing the heterochromatin^50^. We note, while overall trends linked higher heterochromatin organization to greater nuclear stiffness, some mutants deviated from this relationship. R270X and R133C exhibited low HOIs but high stiffness, whereas R255X and T158M showed relatively high or intermediate HOIs yet remained soft. Thus, the HOI and stiffness correlate on average, but local chromatin mechanics can diverge from visible architecture when mutations disrupt DNA-binding affinity, chromatin-bridging motifs, or coupling to chromatin-associated partners^36,50,90–95^. These exceptions indicate that morphological organization alone is insufficient for mechanical reinforcement and underscore that MeCP2-dependent stiffening requires both chromatin structural reorganization and molecular connectivity. Thus, we speculate that the location of the mutation has a direct effect on the local mechanics.

Furthermore, our results showed that the mutant T158M has a strong defect in nuclear stiffening (Fig. 4d, f and Supplementary Fig. 5d, g). This specific mutation is associated with a severe clinical phenotype^89,96^, but, until now, has shown limited impact in traditional in vitro assays^50,97,98^. Further investigation on this specific mutation regarding the effect on the nuclear mechanics could provide critical insights into the mechanisms underlying Rett syndrome and inform therapeutic strategies.

## Methods

### Cell culture conditions

All cells used were deemed mycoplasma free. A list with the main characteristics, as well as reference to the original publications of the cell lines, can be found in Supplementary Table 1.

Murine C2C12 myoblasts were grown in Dulbecco’s Modified Eagle’s medium (DMEM, Cat. No.: 41965039, Gibco) high glucose supplemented with 20% fetal bovine serum (Cat. No.: FBS-22A, Capricorn Scientifics), 110 mg/l sodium pyruvate (Cat. No.: 113-24-6, Sigma Aldrich), 1x L-glutamine (Cat. No.: G7513, Sigma Aldrich) and 1 µM gentamicin (Cat. No.: G1397, Sigma Aldrich), at 37 °C in a humidified atmosphere with 5% CO_2_. For passaging, growth media was aspirated, and cells were briefly washed with 0.02% ethylenedinitrilotetraacetic acid (EDTA, Cat. No.: A5097, AppliChem GmbH) in phosphate buffer saline (PBS), composed of 137 mM NaCl (Cat. No.: 3957, Carl Roth), 2.7 mM KCl (Cat. No.: P9541, Sigma Aldrich), 1 mM Na_2_HPO_4_^.^ 7 H_2_O (Cat. No.: X987, Carl Roth) and KH_2_PO_4_ (Cat. No.: 3904, Carl Roth), before incubation with trypsin-EDTA (Capricorn Scientific, TRY-3B). After 5 min at 37 °C, trypsin was inactivated by the addition of 2× volumes of growth media. In the case of transfection, cells were centrifuged at 1400 rpm for 5 min and resuspended in 100 µl AMAXA M1 solution containing 2–10 µg of plasmid. AMAXA M1 solution was composed of 5 mM KCl (Cat. No.: 7447-40-7, Sigma Aldrich), 15 mM MgCl_2_^.^ 6 H_2_O (Cat. No.: 7786-30-3, Sigma Aldrich), 120 mM of Na_2_HPO_4_/NaH_2_PO_4_ (Cat. No.: 7558-79-4, Sigma Aldrich), and 50 mM mannitol (Cat. No.: 69-65-8, Caesar & Lorentz). Then, cells were electroporated using AMAXA nucleofection system (Lonza, S/N; 10700731), program B-032.

Murine J1 embryonic stem cells (ESC) were grown on feeder-free gelatine-coated culture dishes at 37 °C in a humidified atmosphere with 5% CO_2_. The coating of dishes or coverslips, when necessary, was performed by incubation in 0.2% gelatin from porcine skin (Cat. No.: G2500, Sigma Aldrich) in H_2_O for 15 min at room temperature. ESC growth media consisted of DMEM high glucose supplemented with 15% fetal bovine serum, 1× MEM non-essential amino acid solution (Cat. No.: M7145, Sigma Aldrich), 1× penicillin/streptomycin (Cat. No.: P0781, Sigma Aldrich), 1× L-glutamine (Cat. No.: G7513, Sigma Aldrich), 0.1 mM beta-mercaptoethanol (Cat. No.: 4227, Carl Roth), 1000 U/ml recombinant mouse leukemia inhibitory factor (LIF), 1 µM PD032591 (Cat. No.: 1408, Axon Medchem) and 1 µM CHIR99021 (Cat. No.:1386, Axon Medchem). The media was changed every day. For subculturing, growth media was removed, and cells were briefly washed with PBS before incubation with trypsin-EDTA solution for 5 min at 37 °C. After the incubation, trypsin was inhibited by the addition of 2× volumes of ESC growth media. In case of transfection, cells were centrifuged at 1400 rpm for 5 min and resuspended in 100 µl AMAXA M1 solution containing 2–10 µg of plasmid and electroporated using AMAXA nucleofection system, program A-023.

J1 ESC differentiation was performed by LIF and inhibitors deprivation supplemented with retinoic acid^34,99^. Briefly, 10^3^ cells/cm^2^ were seeded and incubated overnight in ESC growth media. Then, media was removed, cells were briefly washed with PBS, and ESC differentiation media was added. ESC differentiation media had the same composition of ESC growth media, apart from LIF, PD032591, and CHIR99021, which were removed, and the addition of 10 µM retinoic acid (Cat. No.: R2625, Sigma Aldrich). Cells were then incubated at 37 °C and 5% CO_2_ for 7, 14, or 21 days, changing the media every second or third day.

Murine J1 neural stem cells (NSC) were derived from J1 ESC and established as described in the next section. NSCs were grown in plates, slide chambers, or coverslips coated with poly-D-lysine and laminin, prepared in advance, and stored at −20 °C. For coating, plates, slide chambers or coverslips were incubated in 10 µg/ml poly-D-lysine (Cat. No.: P7405, Sigma Aldrich) in H_2_O for 4 h and dried at room temperature for 20 min before an overnight incubation at 37 °C with 5 µg/ml laminin (Cat. No.: L2020, Sigma Aldrich) in ice-cold DMEM-F12 medium (Cat. No.: 56498 C, Sigma Aldrich). NSCs were grown at 37 °C in a humidified atmosphere with 5% CO_2_ in NSC growth media, composed of Euromed-N (Cat. No.: ECL-ECM0883L, Biozol Diagnostica Vertrieb), 1× N-2 supplement (Cat. No.: 17502048, ThermoFisher Scientific), 1× L-glutamine (Cat. No.: G7513, Sigma Aldrich), 1× penicillin/streptomycin (Cat. No.: P0781, Sigma Aldrich), 20 ng/ml murine epidermal growth factor (Cat. No.: 315-09-500UG, Peprotech) and 20 ng/ml murine fibroblast growth factor-2 (Cat. No.: PPT-450-33-500, Peprotech). For passaging, media was removed, and cells were briefly washed with PBS before incubation with accutase (Cat. No.: A6964, Sigma Aldrich) for 3 min. Accutase was then inactivated by the addition of 2× volumes of NSC growth media.

For J1 NSC differentiation to neurons, 2.5 × 10^4^ cells/cm^2^ were seeded in slide chambers or 60 mm plates coated as described above and cultured in NSC growth media for 48 h. Then, the media was changed to neuronal differentiation media composed of 3:1 Gibco Neurobasal media (Cat. No.: 21103049, ThermoFisher Scientific) and DMEM-F12 (Cat. No.: 56498 C, Sigma Aldrich), 0.5x N-2 supplement (Cat. No.: 17502048, ThermoFisher Scientific)), 1x Gibco B-27 supplement (Cat. No.: 12587010, ThermoFisher Scientific), 1× penicillin/streptomycin (Cat. No.: P0781, Sigma Aldrich), 1x L-glutamine (Cat. No.: G7513, Sigma Aldrich), 10 ng/ml murine fibroblast growth factor-2 (Cat. No.: PPT-450-33-500, Peprotech), 20 ng/ml brain derived neurotrophic factor (BDNF, Cat. No.: AF-450-02-10UG, Peprotech). Differentiation media was changed every 3-4 days for 14-21 days to obtain differentiated neurons.

### Generation of J1 ESC MeCP2 KO cell line

To generate J1 ESC knockout for the *Mecp2* gene (MeCP2 KO), a MIN strategy was followed^100^. First, a J1 ESC containing the MIN tag in the exon 2 of the *Mecp2* gene (J1 ESC MIN-MeCP2) was generated by CRISPR/Cas9 (Supplementary Fig. 2a). To do so, a plasmid containing the template for the guide RNA (CACCGTCAGAAGACCAGGATCTCCA) as well as the Cas9 was generated. DNA oligos (forward and reverse) coding for the guide RNA (Supplementary Table 2) were annealed by mixing 100 µM in NEB 4 buffer, incubated at 95 °C for 5 min, and then cooled at room temperature for 10 min. The annealed DNA, together with the pSpCas9(BB)-2A-Puro (Addgene #62988, Supplementary Table 3), was incubated with BpiI (ThermoFisher Scientific) and 30 U T4 DNA ligase (Cat. No.: M0202, New England Biolabs) in T4 DNA ligase buffer, in 55 cycles of 5 min 37 °C and 5 min 20  °C followed by 60 min at 37 °C and 10 min at 95 °C and the mix used to transform TOP10 *Escherichia coli* (Cat. No.: C404003, ThermoFisher Scientific). The resulting plasmid was verified by DNA sequencing. J1 ESC were then transfected with the previously constructed plasmid together with the single-stranded repair template containing the MIN tag, which was chemical synthetized (CTTCTTTGTCCTCCTTCTTGTCTTTCTTCGCCTTCTTAAACTTCAGTGG CTTGTCTCTGAGGCCCTGGAGATCCTGGGTTTGTACCGTACACCACTGAGACCGCGGTGGTTGACCAGACAAACCGTCTTCTGACTTTTCCTCCCTGAAGTATTAAACAAATATGTAAGTATTACAGAGAACACAGCTGTCTGCACAGTAG). Cells were seeded onto 365-well plates for selection with 10 µg/ml puromycin (Cat No.: ant-pr-1, InvivoGen) for 2 days. The presence of the MIN tag was then checked by genomic DNA isolation followed by PCR (Supplementary Fig. 2b) with the oligos described in Supplementary Table 2, and the DNA from positive clones was sequenced to confirm the correct MIN tag integration in exon 2 of the *Mecp2* gene locus (Supplementary Fig. 2b).

Subsequently, the MeCP2 KO was generated by introducing a stop codon in the MIN tag. This was achieved by transfecting the J1 ESC MIN-MeCP2 with a plasmid coding for the Bxb1 recombinase (pCAG-NLS-Bxb1, Addgene #65625, Supplementary Table 3) together with a plasmid containing the attB site, followed by mCherry cDNA and a stop codon (pattB-Cherry-Stop-puro, Addgene #65529, Supplementary Table 3). Transfected cells were grown in 365-well plates and selected by genomic DNA screening PCR (Supplementary Fig. 2c). The cell line generated was further characterized by verifying its ability to form colonies (Supplementary Fig. 2d), its pluripotency by staining for the Oct4 marker (Supplementary Fig. 2i, j), its self-renewal by labeling and staining for proliferating cells with EdU (Supplementary Fig. 2k). To confirm the successful removal of MeCP2, due to the low levels of MeCP2 in ESC (Supplementary Fig. 2e), both wt and MeCP2 KO ESC were differentiated into neural lineage by LIF deprivation (Fig. 3a and Supplementary Fig. 2f), confirmed by NeuN staining (Supplementary Fig. 2h), to quantify the MeCP2 levels in the differentiated wt cells while not increasing in the MeCP2 KO cells (Supplementary Fig. 2f, g).

### J1 NSC cell line generation

J1 ESC wt or J1 ESC MeCP2 KO were cultured at 37 °C in a humidified atmosphere with 5% CO_2_ in 0.2% gelatin-coated T25 flask in Gibco KnockOut Dulbecco’s modified Eagle’s medium (Cat. No.: 10829018, Thermo Fisher Scientific) supplemented with 15% KnockOut serum replacement (Cat. No.: 10828028, Thermo Fisher Scientific), 1× MEM non-essential amino acid solution, 1× penicillin/streptomycin (Cat. No.: P0781, Sigma Aldrich), 1× L-glutamine (Cat. No.: G7513, Sigma Aldrich), 0.1 mM beta-mercaptoethanol and 1000 U/ml recombinant mouse LIF. Cells were passaged multiple times by briefly washing with PBS and then incubated with accutase for 3 min at 37 °C until they grew as monolayers (Supplementary Fig. 2a, left panel). Then, 4 × 10^4^ cells/cm^2^ were seeded in 0.2% gelatin-coated 6-well plates and cultured in neurobasal media and DMEM-F12 (1:1) medium supplemented with 0.1 mM beta-mercaptoethanol, 0.5× N-2 supplement (Cat. No.: 17502048, ThermoFisher Scientific), 0.5× B-27 supplement (Cat. No.: 12587010, ThermoFisher Scientific), 1x GlutaMAX supplement (Cat. No.: 35050038, Thermo Fisher Scientific) and 1× penicillin/streptomycin (Cat. No.: P0781, Sigma Aldrich) for up to 7 days at 37 °C in a humidified atmosphere with 5% CO_2_ until maximal confluence was reached. Then, cells were dissociated with accutase, reseeded in non-coated T25 flask, and incubated in NSC growth media at 37 °C and 5% CO_2_ for 2–4 days allowing the formation of neurospheres of 100–200 µm diameter (Supplementary Fig. 4a, middle-left panel). Once they reached this diameter, neurospheres were transferred to a 15 ml tube using a wide bore tip and spinned down at 500 rpm, then resuspended gently in fresh NSC media. Then, neurospheres were seeded in poly-D-lysine/laminin-coated T25 flasks, where neurospheres were attached to the dish and started the differentiation to NSC. Once confluence in the T25 flask was reached, cells were passaged using accutase and split at 1:2 ratio into new culture dishes. Stable NSC cell lines were established after 15 passages (Supplementary Fig. 4a, middle-right panel). NSCs were validated using Pax6 marker immunostaining (Supplementary Fig. 4b), and their self-renewal ability was confirmed by labeling and staining for proliferating cells with EdU (Supplementary Fig. 4c). The absence of MeCP2 protein was again confirmed by immunostaining with anti-MeCP2 specific antibodies (Supplementary Fig. 4e), as well as by western blot (Supplementary Fig. 4f).

### Cell immunostaining and EdU click-it reaction

For immunostaining, cells were grown on appropriately coated coverslips or slide chambers and differentiated (if required) as described above. Cells were washed twice with PBS and then fixed using either 3.7% formaldehyde for 10 min or ice-cold methanol for 6 min, depending on the antibodies used, as stated in Supplementary Table 4. After three times washing with PBS, cells were permeabilized with 0.5% Triton X-100 (Cat. No.: 10670, LS Laborservice) in PBS and washed three times with 0.01% Tween-20 (Cat. No.: 9127.1, Carl Roth) in PBS (TPBS). Then, cells were incubated in a blocking solution composed of 1% BSA in PBS for 20 min. After blocking, samples were incubated with primary antibody, undiluted or diluted in blocking solution as stated in Supplementary Table 4 for 2 h. In each immunostaining assay, a secondary control was performed following the same procedure but skipping the primary antibody incubation. Non-bound antibodies were washed three times with TPBS before adding the secondary antibody diluted in PBS as stated in Supplementary Table 4, followed by another three washing steps with TPBS. Samples were then counterstained with 1 µg/ml of the DNA dye DAPI, washed twice in PBS and once in distilled H_2_O, dried, and mounted on a slide into a drop of Mowiol mounting media composed of 13% Mowiol 4-88 (Cat. No.: 81381, Sigma Aldrich), 33% glycerol (Cat. No.: G9422, Sigma Aldrich), 2% 1,4-diazabicyclo-[2.2.2]octane (Cat. No.: D2522, Sigma Aldrich) and 133 mM Tris-HCl pH 8.5 (Cat. No.: 93362, Sigma Aldrich).

For EdU (Cat No: 7845.1, Carl Roth) incorporation detection, cells were grown on appropriately coated coverslips and pulsed with 10 µM EdU for 20 min. EdU can only be integrated into the genome during active DNA replication. A click-it reaction, following the manufacturer’s protocol, was performed to link the EdU with a dye: 6-carboxyfluorescein (Cat. No.: 7806.2, Carl Roth) or Eterneon-Red 645 (Cat. No.: 1Y73.1, Carl Roth). DNA was counterstained with 1 µg/ml of DAPI for 15 min, and cells were mounted in a glass slide with Mowiol mounting media.

### Flow cytometry and GFP-intensity based categories

Cells were transfected as described above and one untransfected plate was grown in the same conditions. Cells were harvested 20 h after transfection, resuspended in PBS, and separated according to their transfection level by fluorescence-activated cell sorting (FACS) on a S3 Cell Sorter (Supplementary Table 5) into three gates^35,44^. Briefly, cells were exposed to a 488 nm laser, and intensity was measured after a 525 ± 30 nm emission filter. Cells were plotted against log10 of the sum intensity. Intensities were grouped into bins with a value calculated as the difference between the maximum of the transfected and untransfected samples divided by 42. Then, gates were defined as follows: negative (values lower than 8×bin), low expressing (values within 13×bin and 22×bin), and high expressing (values within 24×bin and 33× bin).

### Nuclei purification

We modified a protocol described previously^101^. Cells were dissociated from the plate by trypsin-EDTA solution and resuspended in the correspondent growth media. To remove the media, cells were centrifuged at 1400 rpm for 5 min, and the supernatant was discarded. Then, pellets were resuspended in ice-cold PBSN, composed of 0.1% Nonidet P-40 substitutive (Cat. No.: 74385 Sigma Aldrich) in PBS, triturated by continuous pipetting, and pelleted by short centrifugation at high speed (13,000-16,000 rpm) for 20 s. When needed, pellets were then resuspended in PBSN containing 1 µg/ml DAPI and incubated at room temperature for 15 min or, alternatively, resuspended in PBSN without incubation time. A second centrifugation at high speed for 20 s was done and the resulting pellet was resuspended in buffer A2, composed of 20 mM Triethanolamine-HCl (Cat. No.: T-1377, Sigma Aldrich) buffer, 30 mM KCl, 10 mM MgCl_2_ • 6 H_2_O, 0.25 M sucrose (Cat. No.: 4661, Carl Roth) and 0.1 mM phenylmethylsulfonyl fluoride (Cat. No.: 6367, Carl Roth). In this buffer, nuclei were kept at 4 °C for a maximum of one week.

### Plasmids

All plasmids used, and their characteristics, are listed in Supplementary Table 3.

The plasmids pEG-MeCP2 R270X and pEG-MeCP2 R294X were generated using a Q5 directed mutagenesis from the pc1208 (pEG-MeCP2) following the standard protocol described by the manufacturer (New England Biolabs, E554S). The primer pairs flanking the regions to be deleted are listed in Supplementary Table 2. The mutation was added by DNA polymerization reaction, and the reaction product was then incubated in a buffer with kinase, ligase, and DpnI to phosphorylate and ligate the newly generated plasmid containing the mutation while digesting away the template. Finally, *E. coli* TOP10 cells were transformed with the reaction mix. Plasmids were then sequenced to confirm the mutation.

### Atomic force microscopy

For each experimental group, nuclei were seeded on partially dehydrated agarose pads prepared from 0.5% agarose (Cat. No.: A9539, Sigma Aldrich) in PBS, evenly distributed on 50 mm glass coverslips. Once the buffer in which the nuclei were resuspended had partially drained into the agarose, fresh buffer A2 was added on top for the measurements. Samples were selected using bright-field microscopy on the AFM setup, positioning the cantilever tip over regions containing islands of nuclei (with ≥5 nuclei in each frame).

We employed Force-Volume (F–V) mode, which involves static loading rather than oscillatory measurements. Consequently, the measured modulus reflects contributions from both shear and compressive deformation, though compressive effects are limited due to material incompressibility. Regardless of the time scale, local indentation captures both deformation types as the tip indents the material until the setpoint force is reached. The reported modulus is not an absolute measure but is used for comparative analysis of relative differences in nuclear mechanics under varying chromatin reorganization conditions. F–V mapping was carried out using a Nanowizard II AFM (JPK Instruments) coupled with a Zeiss Axio Observer Z1 optical microscope (Supplementary Table S5). This technique employs a linear ramp method, where a complete force‒distance (F‒D) curve is recorded at each pixel. Soft cantilevers with nominal spring constant of 0.06 N/m (triangular-shaped microlevers, SNL-D, nonconductive sharp silicon nitride from Bruker) were employed for measuring the mechanical properties and performing spectroscopy of the cells. These cantilevers have a nominal flexural resonance frequency of 18 kHz in air and a tip radius of 10 nm. The inverse optical lever sensitivity of the AFM system was determined by recording a single F‒D curve on the glass substrate (coverslip) of the nuclei in fresh buffer A2. Additionally, the cantilever spring constant was calibrated using the thermal noise method^102^. For comparative analysis of the stiffness values obtained from the control measurements through F‒V mapping in this study, the following experimental parameters were set: the trigger point was adjusted to 2 nN to ensure a large indentation range of approximately 1 µm. An upper indentation threshold of 1 µm was used to ensure a high signal-to-noise ratio and to maintain a stable, near-linear regime of the force–indentation response. This value served as both the physical indentation limit and the upper boundary for elastic modulus fitting, as illustrated by the representative profiles in Supplementary Fig. 3n. Furthermore, the z-length was set to 3 µm, the extension time was 90 s, the maximum sample rate was 16,000 Hz, and the grid size was 30 × 30 µm² with 64 × 64 points. The entire force-volume map was subsequently processed using a self-written MATLAB script to generate a stiffness distribution as introduced by ref. ^103^ and demonstrated in our previous studies^104,105^.

Force–distance (F–D) curves were first corrected for baseline tilt by fitting a line to the non-contact region (50–99% of z-piezo displacement, where the cantilever was fully withdrawn). The contact point was defined as the position where the cantilever deflection first deviated systematically from the baseline during approach. The indentation depth δ was calculated as the z-piezo displacement minus cantilever deflection. These force-indentation (F–d) curves were then fitted to the Derjaguin-Muller-Toporov (DMT)-type contact model^106^ adapted for a conical indenter, to produce local elasticity (*E*) maps, according to Eq. 1. For each curve, the first 8 nm after contact were discarded to minimize tip–surface detection artifacts, and the subsequent 1 µm of indentation was used for fitting.

1 $${F}_{0}=\frac{2}{\pi \,}\cdot \frac{E}{1-{\upsilon }^{2}}\cdot \tan \theta .\,{\delta }^{2}-2\pi {aW},$$

being ${F}_{0}$ the force, *ν* the sample’s Poisson ratio (: lateral to axial strain, ranges from −1 to 0.5, with *ν* = 0.5 representing the incompressible limit), *θ* is the cone half-angle, *δ* is the indentation depth, $a=\delta \tan \theta$ the contact radius, and $W$ is work of adhesion. To correct for finite-thickness support provided by the agarose substrate, we applied the BECC model^41^, implemented in MATLAB, as given in Eq. 2:

2 $$F={F}_{0}\,[1+0.721(\frac{a}{h})+0.650{(\frac{a}{h})}^{2}+0.491{(\frac{a}{h})}^{3}+0.225{(\frac{a}{h})}^{4}],$$

where $h$ is the sample thickness. Local modulus values extracted from 4096 curves per nucleus were compiled into elasticity maps, and the global nuclear modulus was obtained by averaging all local values.

The global elastic modulus for each nucleus was calculated by averaging the cumulative local elastic modulus values obtained from F–D curves measured across the nuclear surface. In total, 4096 F–D curves were acquired per scan area, with approximately 300 curves corresponding to each individual nucleus. This resulted in a minimum of ~4500 force curves analyzed per experimental group. Such sampling density ensures statistical robustness and supports the reliability of the reported elastic modulus values.

Nuclear volume measurements from AFM images were performed using the built-in volume tool in Gwyddion 2.54 (Department of Nanometrology, Czech Metrology Institute, Czech Republic), which integrates height over the masked nuclear area. Raw height data were used for volume measurements to preserve the true topography of the nucleus without distortion from background correction. As the nuclei are seeded on a porous agarose surface and may be partially trapped within the pores, the measured volume reflects only the portion protruding above the surface. We note that this may result in an underestimation of absolute nuclear volume. Furthermore, to ensure an accurate baseline, the mask was slightly extended by 1 pixel beyond the nuclear boundary onto the agarose pad to define a consistent minimum height for volume integration.

### High throughput microscopy

Cells were immunostained as described above. For image acquisition, a Nikon-CREST widefield microscope was used (Supplementary Table 5). During imaging, the acquisition conditions (exposure time, optical gain, and laser power) were kept constant between samples in the channel containing the protein of interest (POI), while the counterstaining channel (generally DAPI) conditions were modified to get the best segmentation of individual nuclei in each field, unless DAPI was used for normalization of the data.

Analysis of the images was done in ImageJ. First, the DAPI channel was used for segmenting the nuclei. Standard processing of Gaussian blur (sigma of 5 or 10 pixels if 20X or 40X objective was used, respectively) followed by a background subtraction (rolling background of 20 or 50 pixels, in 20X or 40X images, respectively) and Otsu auto-threshold. The nuclei were defined as ROI using the Analyze Particle function, only restricting the edges, and checked manually to remove or modify nuclei that were not properly segmented.

Once the ROIs in the nuclei were determined, the area and the total intensity (IntDen) were calculated for each channel. For comparing between conditions, intensity values from at least three biological replicates were merged and compared in a violin plot. Statistical differences were calculated using *F*-test assuming equal variance between samples.

### Acquisition and analysis of 3D samples

Z-stacks were taken following Nyquist sampling in a Leica TSC SPE-II confocal point scanner microscope (Supplementary Table 5). For the 3D analysis using ImageJ 3D suite, individual cells were segmented manually and then processed to: (i) get the nuclear volume, by applying a 3D median filter with 3 × 3 × 2 pixels (xyz) followed by Otsu threshold segmentation; (ii) get the heterochromatin compartments, by using deconvolution^107^. The resulting image was thresholded and applied a skeletonize to generate a seed that was then used for the 3D spot segmentation of the 3D suite, together with the deconvolved image. The resulting spots were then filtered by the nuclear mask. Volumes and numbers were then taken from the respective masks using 3D measurements of the 3D suite.

### RNA-seq and ChIP-seq

We used published datasets for the RNA-seq and ChIP-seq. For RNA-seq, the raw counts of genes in RNAseq analysis were downloaded from the GEO repository. We used the dataset GSE140054^108^ for the comparison of wt and MeCP2 KO, specifically the samples GSM4152139-GSM4152148, which correspond to cortex of wt and MeCP2 KO mice, 5 replicates each. Additionally, we used the dataset GSE83474^109^ to compare the wt MeCP2 versus the Rett mutations, using the samples GSM2203994-GSM2204004, which correspond to 4 replicates of excitatory neurons of a pool of 2–3 mice (six weeks-old) for Mecp2^wt/Y^, Mecp2^R106W/Y^ and Mecp2^T158M/Y^. The differential DESeq2 Plots were visualized using RStudio (R version 4.4.0, Version 2024.04.1 + 748 (2024.04.1 + 748)).

Sratoolkit (version 2.11.0) was used to download the ChIP-seq datasets from the GEO-database (Gene Expression Omnibus, https://www.ncbi.nlm.nih.gov/geo/), specifically the dataset GSE90704^110^ and the samples G2410973-GSM2410978, which correspond to brain cortex of 6 weeks-old wt mice. Read quality was checked using the FastQC program (version 0.11.9). When necessary, Trimmomatic (version 0.36) was used to remove reads of poor quality. Next, Bowtie2 (version 1.3.1) was used to align the reads to the mouse genome (mm10 genome assembly, https://hgdownload.cse.ucsc.edu/goldenpath/mm10/bigZips/), and Samtools (version 1.10) was used to generate BAM files. Samtools were also used to merge the 3 replicates of MeCP2 Chip-seq/input correspondingly. The input signal was subtracted from the Chip-seq signal using deepTools (version 3.5.1_singularity), command bamCompare, using the option subtract. DeepTools were also used to plot profiles around the TSS of genes identified as unchanged/ changed in R106W and changed in T158M.

### Western blots

First, the cells were trypsinized and centrifuged for 5 min (2000 rpm at 4 °C). Supernatant was removed. The pellet was resuspended in 200 µL of lysis buffer (20 mM Tris HCl (pH 8), 150 mM NaCl, 1.5 mM MgCl2, 0.4% NP-40, 0.2 mM EDTA, and protease inhibitors 1 mM AEBSF (4-(2-Aminoethyl) benzyl sulfonyl fluoride hydrochloride, Cat. No.:A1421.0100, VWR, Radnor, PA, USA), 1 mM E64 (Cat. No.: E3132, Sigma-Aldrich, St Louis, MO, USA), 1 nM Pepstatin A (Cat. No.: 77170, Sigma-Aldrich, St Louis, MO, USA), PMSF (10 µM, Sigma-Aldrich, St. Louis, MO, USA/Solarbio; catalog #P8340) and AEBSF (1 mM, AppliChem, Darmstadt, Germany). Homogenization of the cell extracts was performed using a syringe and 21 G needles, with 25 strokes per sample, while maintaining the samples on ice during the strokes. This was followed by an incubation on ice for 25 min. During this incubation, vortex was applied to the samples every 10 min. After incubation, loading buffer 6x SDS (400 mM DTT, 200 mM Tris/HCl pH 6.8, 8% SDS, 0.4% bromophenol blue, and 40% glycerol) was added to the samples, which were subsequently boiled at 95 °C and separated on 8% SDS-PA (sodium dodecyl sulfate–polyacrylamide) gels. The SDS-PAGE and Western blotting experiments were performed as in refs. ^111,112^ small variations. Four microliters of protein ladder marker (MWP06 BlueEasy Prestained Protein Marker, Nippongenetics) were loaded into the gel. Transference of protein from the gel to the membrane (Nitrocellulose, GE Healthcare, München, Germany) was performed in a semi-dry blotting chamber (Bio-Rad Laboratories) for 50 min at 25 V, followed by Ponceau S staining and colorimetric imaging. Blocking was performed for 1 h in low-fat milk 3% dissolved in 1x PBS at room temperature. After blocking, membranes were incubated with primary antibodies diluted in the blocking buffer overnight at 4 °C in rotation. Following primary antibody incubation, the membranes were washed 3 times for 10 min each with 1x PBS + 0.02% Tween-20 before incubation with the secondary antibodies for 1 h and washing as described before. For visualization of the bands, horseradish peroxidase (HRP) or fluorescent-conjugated secondary antibodies were used. All the characteristics and dilutions of primary and secondary antibodies and dilutions used are described in Supplementary Table 4. To develop the membranes, Pierce™ ECL Western Blotting Substrate was used (Cat. No.: 32209, ThermoFisher Scientific, Waltham, MA, USA). The Amersham AI600 Imager with a CCD camera (GE Healthcare, Chicago, IL, USA) was used to image immunoreactive bands and Ponceau S staining. For a better composition of the figures and due to space restrictions, cutouts of the membranes were made. Unprocessed scans for all the blots are provided with the data sets uploaded to TUdatalib.

### Statistics and reproducibility

The representation of the data as violin plots, as well as the probability density functions (PDF), was done in MATLAB R2021a update 8 (6.10.0.2198249), using GAVI, a self-written script. The script is available in the TUdatalib (see data availability section). GAVI collects the data from a “.csv” (comma-separated values) file, which contains the conditions in individual columns and represents them as individual probability density function graphs and/or violin plots. Additionally, it performs a Gaussian Mixture Model (GMM) analysis, in which the model with lesser Bayesian Information Criteria is selected as the best fitting, to divide the data into populations, providing the mean value of each population as well as the percentage of the total measurements that would fall into the respective populations. Lastly, it performs a two-sided t-test with the null hypothesis of equal means with equal variance between all the datasets to quantify the significance of differences between the data.

For the elastic modulus PDF representations, the size of the bars is 0.05 log (Pa). The use of logarithms in these graphs was chosen to enhance visibility, but neither the differences nor the GMM analysis changed when using the raw data.

In the violin plots, the x spread represents the frequency of the data in the corresponding y, the gray box represents the 1st and 3rd percentiles, the white dot the median, and the whiskers the standard deviation.

To assess relationships between nuclear morphology, chromatin organization, and mechanical properties, non-parametric and regression analyses were performed in RStudio (version 2024.12.1 + 563). To test whether nuclear size contributes to mechanical stiffness, two-tailed Spearman’s rank correlation coefficients (*ρ*) were computed between nucleus volume (µm³) and the mean elastic modulus (Pa) across individual nuclei (10 per condition) using the cor.test() function with method = “spearman”. This non-parametric test was chosen to capture monotonic associations independent of data normality.

To evaluate the relationship between chromatin organization and nuclear stiffness, the heterochromatin organization index (HOI) was calculated for each condition as the ratio of the mean heterochromatin compartment volume (normalized to nuclear volume) to the mean number of compartments per nucleus. The HOI was log₁₀-transformed to account for its exponential range. Spearman’s rank correlations were then performed between log (HOI) and the weighted stiffness contributions of the soft, mid, and stiff nuclear fractions, derived from GMM of AFM elastic modulus distributions. Weighted stiffness values were computed by multiplying the fraction proportion by its respective mean elastic modulus. Given the limited number of conditions, correlations were interpreted qualitatively.

In addition, linear regression analyses were conducted using the lm() function in RStudio to evaluate how heterochromatin organization (log₁₀HOI) predicts stiffness parameters. The slope (*β*), coefficient of determination (*R*²), and *p* value were reported to describe the strength and direction of association.

No statistical methods were used to predetermine sample size. Investigators were blinded to experimental conditions and outcomes during the AFM measurements and analyses. All cells analyzed displayed the representative phenotypes and morphological features shown in the corresponding figures, and no data points were excluded from analysis.

### Rett mutation elastic modulus screening

In the screening of the Rett mutations, samples were transfected as described in Cell culture conditions, and transfection frequency was determined. Only samples with transfection frequencies of 70–75% were used. Nuclei were then purified, and the mixture of transfected and untransfected nuclei was measured in atomic force microscopy. Accordingly, to compare the effect of Rett mutants to wild type MeCP2, the latter was done in the same conditions, i.e., no a priori selection of cells was performed. Hence, the data variability in Fig. 4 reflects the higher variability on protein levels when compared to the results shown in Fig. 2, where cells were sorted by protein levels.

For the k-means clustering analysis using MATLAB, two components of the distribution of the elastic modulus were taken into account. In the first place, a Gaussian mixture model for three populations (based on the results of Fig. 2) was applied to the individual samples, and the mean of the populations was extracted. Next, all data were pooled to define three common populations, and the individual samples were assigned to these populations to determine the relative weight (percentage) of each population. To minimize bias in distance calculation arising from differences in the values, the z-score function was used to normalize the data. Euclidean distances were then calculated from the normalized data, and clustering was performed using the Ward linkage algorithm, represented as a dendrogram. The number of clusters was defined based on the inconsistency coefficient.

## Supplementary information

Supplementary information

## Data Availability

The raw data and analyses that support the findings of this study are available in the TUdatalib repository with the identifier 10.48328/tudatalib-1590. All datasets and analysis scripts can be directly accessed and downloaded from this repository.

## Code availability

All custom MATLAB scripts and ImageJ macros used in this study are publicly available in the TUdatalib repository at 10.48328/tudatalib-1590. The deposited files match the versions used in this work and include the parameters necessary for reproducibility. There are no access restrictions.
