# Supplementary material for: MeCP2-driven chromatin organization controls nuclear stiffness
Source: Commun Biol. 2025 Dec 8;9:60. doi: 10.1038/s42003-025-09328-6 (PMC12804834; doi:10.1038/s42003-025-09328-6)
Supplement: Supplementary file 1 — Supplementary information [file 42003_2025_9328_MOESM1_ESM.pdf]

MeCP2-driven chromatin organization controls nuclear stiffness

*Hector Romero[#1], Anahid Amiri[#1,2], Maruthi K. Pabba[1], Hui Zhang[1], Veronika Berg[1], Maria Arroyo[1], Paulina Prorok[1], Andreas Zhadan[1], Marah Mahmoud[1], Nina Trautwein[3], Bodo Laube[3], Christian Dietz[2], Robert W. Stark[2] and M. Cristina Cardoso[*1].*

[1] Cell Biology and Epigenetics, Department of Biology, Technical University of Darmstadt, Darmstadt, Germany.

[2] Institute of Materials Science, Technical University of Darmstadt, Darmstadt, Germany.

[3] Neurophysiology and Neurosensory Systems, Department of Biology, Technical University of Darmstadt, Darmstadt, Germany.

[#] These authors have contributed equally.

[*] Correspondence: cardoso@bio.tu-darmstadt.de

Supplementary Materials

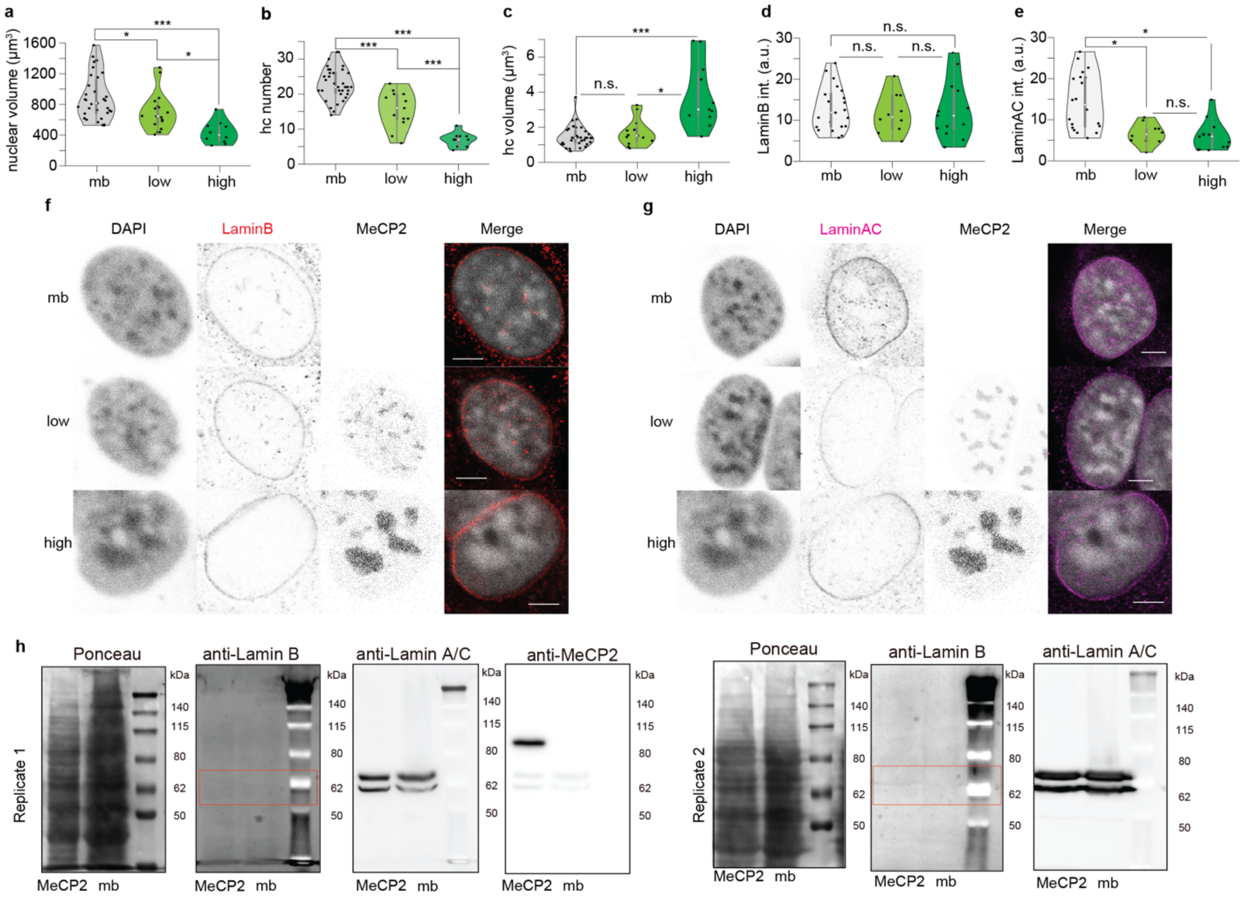

Supplementary Figure 1. Supplementary information to Figure 2.

a-c. 3D confocal analysis of the C2C12 myoblast. Cells were transfected with pGFP-MeCP2, seeded in glass cover slides and cultured overnight. Then, cells were fixed with formaldehyde, stained with DAPI and mounted in Mowiol. 3D stacks were taken in confocal and analyzed in FIJI. ~500 cells were imaged in 2D to calculate the normalization curve and define the transfection categories. The results are summarized in violin plots for the nuclear volume (a), the number of heterochromatin compartments per cell (b) and the average heterochromatin volume per cell (c). In the violin plots, the x spread represents the frequency of the data in the corresponding y, the gray box represents the 1st and 3rd percentiles, the white dot the median, and the whiskers the standard deviation. To assess the significance, a 2-sided t-test was performed to calculate a p-value. Only significant differences are shown. *: p-value < 0.05; **: p-value < 0.001; ***: p-value < 0.0001. **d-g.** Immunofluorescence analysis of LaminB and LaminAC. Quantification of levels of Lamin B (d) and LaminAC (e) by confocal imaging. Representative images are provided to assess the possible differences in LaminB (f) or LaminAC (g) nucleoskeleton. Scale bar is 5 μm. **h.** Western blot analysis to address the total levels of LaminB and LaminAC in the C2C12 untransfected (mb) versus transfected with pGFP-MeCP2 (MeCP2).

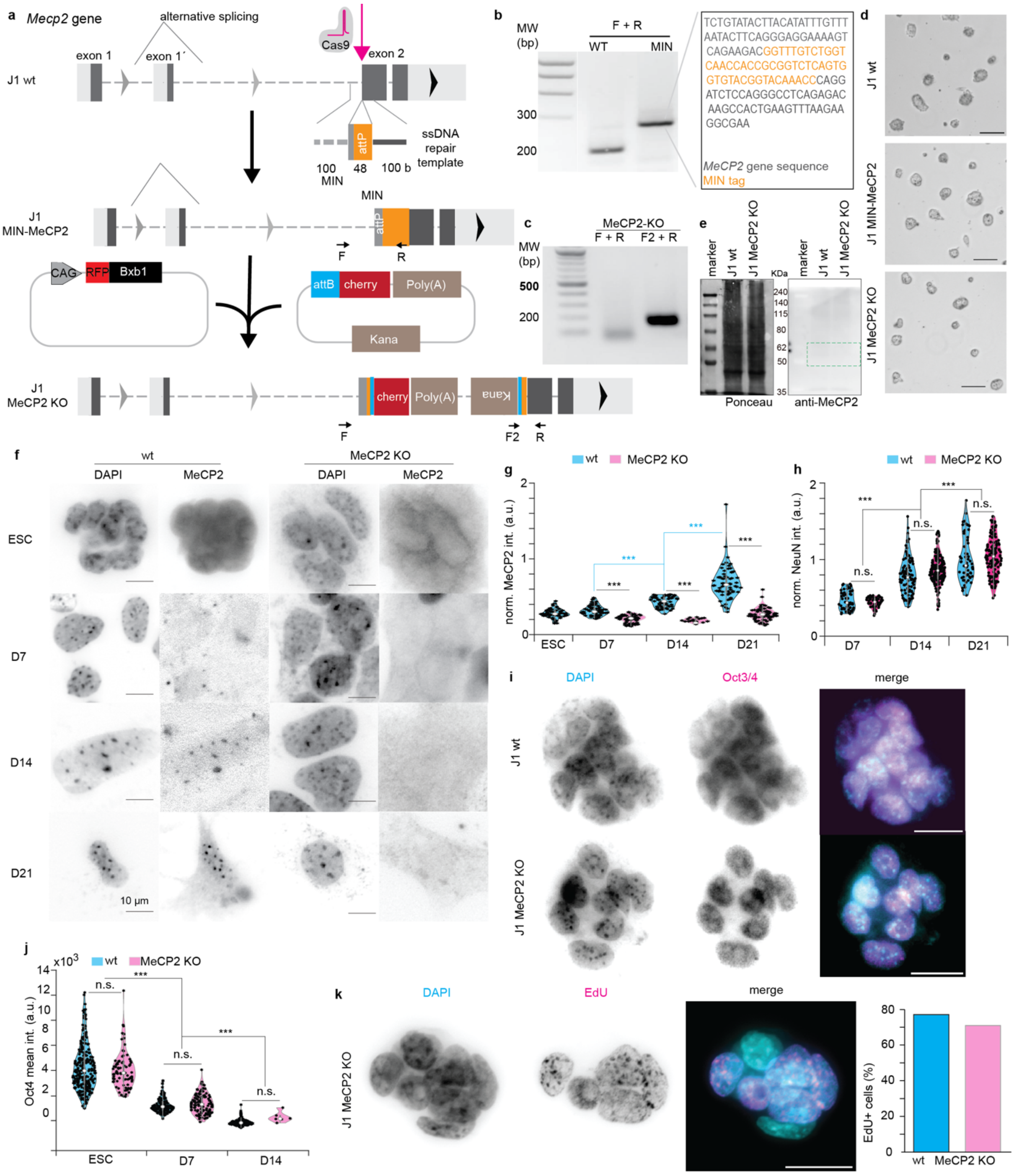

Supplementary Figure 2. Generation and characterization of J1 ESC MeCP2 KO.

a. Overview of the generation of J1 ESC MeCP2 KO cell line. First, a MIN tag that contains an attP site was introduced by CRISPR-Cas9 into the exon 2 (common for both MeCP2 isoforms). Once inserted, a transfection with a plasmid containing the recombinase Bxb1 and the plasmid containing an attB site followed by a mcherry gene and a polyA signal. Bxb1 recognizes attP and attB sites to recombine, therefore inserting the mcherry and the polyA signal after exon 1 and truncating the MeCP2 expression. F, F2 and R represent the location of the primers used for the screening of positive clones. **b.** Confirmation of the MIN integration by screening genomic PCR followed by sequencing. **c.** Screening PCR for the insertion of the mCherry-polyA plasmid in the exon 2. The relatively large

genomic fragment generated by the pair of primers F-R is not efficiently amplified during the elongation time used for the PCR, while the expected DNA band at 204 bp from the primer pair F2-R, being shorter requires less time of synthesis and is, thus, amplified. **d.** Overview of colonies of J1 wt, J1 MIN-MeCP2 and J1 MeCP2 KO ESC. Scale bar: 100 µm. **e.** Confirmation of the MeCP2 KO at protein level. Western blot analysis of ESC (e) did not yield any signal as wt cells have undetectable levels of the protein (marked with a dotted green line where MeCP2 is expected). Ponceau was used as total protein loading control (left panel). **f-h.** ESC was differentiated to neuronal fate by removing the Leukemia Inhibitor Factor (LIF) and the two inhibitors (2i) known to maintain pluripotency of the ESC. MeCP2 levels were addressed by immunostaining with anti-MeCP2 antibody (f), and nuclear MeCP2 intensity (or lack thereof) was quantified (g), together with the quantification of the neuronal marker NeuN (h). Both MeCP2 and NeuN were normalized to the DNA (DAPI) intensity. Scale bar in representative images; 10 µm. **i-j.** Confirmation of the pluripotency by immunostaining with the pluripotency marker Oct4, as shown in the representative images. Scale bar 20 µm. (i). A quantification during the first stages of the LIF removal differentiation was done in parallel to the wt cells by high throughput microcopy (j). **k.** Visualization of the replication potential of the MeCP2 KO ESC by EdU incorporation. Scale bar: 20 µm. >400 cells in two biological replicates were counted. The percentage of positive EdU cells was compared to previous results in J1 wt cells [99]. In the violin plots, the x spread represents the frequency of the data in the corresponding y, the gray box represents the 1st and 3rd percentiles, the white dot the median, and the whiskers the standard deviation. Significance was defined based on the p-values obtained from a two-sided t-test. n.s.: non-significant (p-value > 0.05) *: p-value < 0.05; **: p-value < 0.001; ***: p-value < 0.0001.

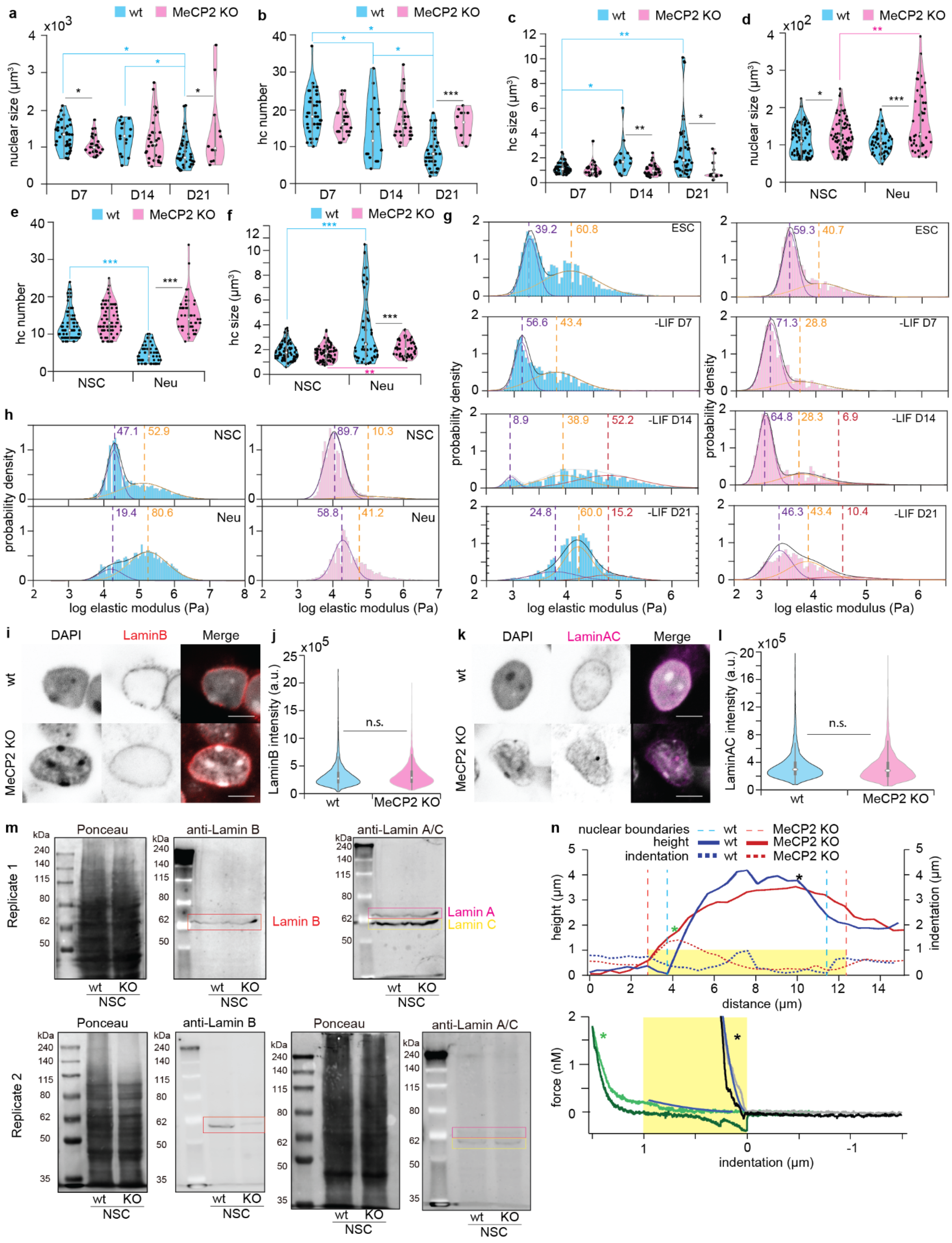

Supplementary Figure 3. Supplementary information for figure 3.

a-c. 3D confocal analysis of wt and MeCP2 KO cells in the different stages of the LIF deprivation fixed with formaldehyde and stained with DAPI, representing the nuclear volume (a), heterochromatin compartments in each cell (b) and average heterochromatin volume per cell (c). The averages and 95% confidence intervals of these data are plotted in Figure 3b. **d-f.** 3D confocal analysis of wt and MeCP2

KO cells in the different stages of the NSC differentiation fixed with formaldehyde and stained with DAPI, representing the nuclear volume (d), heterochromatin compartments in each cell (e) and average heterochromatin volume per cell (f). The averages and 95% confidence intervals of these data are plotted in Figure 3b. In the violin plots, the x spread represents the frequency of the data in the corresponding y, the gray box represents the 1st and 3rd percentiles, the white dot the median, and the whiskers the standard deviation. In all violin plots, a 2-tailed t-student assuming equal variance was used to calculate the p-value, comparing the different categories for the same cell and the different cells for each category. Only significant differences are shown. *: p-value < 0.05; **: p-value < 0.001; ***: p-value < 0.0001. Significance was defined based on the p-values obtained from a two-sided t-test. n.s.: non-significant (p-value > 0.05) *: p-value < 0.05; **: p-value < 0.001; ***: p-value < 0.0001. **g-h.** The histograms of the elastic modulus values from the different stages of neural differentiation. A Gaussian mixture model was added, showing the mean value (dashed lines) and the weight (numbers on top of the panel, in %) for each population. The third population was removed and readjusted to two populations in samples where the third population overlapped one of the existing two or its weight was under 1%. The differentiation was done directly from ESC with LIF deprivation (g) or from stable NSC cell lines (h). **i-j.** LaminB immunostaining analysis for wt and MeCP2 KO NSCs fixed with methanol. Representative images (i) were used to define the (absence of) changes in morphology of the lamin nucleoskeleton. A high-throughput analysis (j) was performed to assess the total lamin levels per nucleus. **k-l.** LaminAC immunostaining analysis for wt and MeCP2 KO NSCs fixed with methanol. Representative images (i) were used to define the (absence of) changes in morphology of the lamin nucleoskeleton. Scale bar: 5 μm. A high-throughput analysis (k) was performed to assess the total lamin levels per nucleus. **m.** LaminB and LaminAC western blot analysis. The size of expected band size is represented with a rectangle (red: Lamin B; magenta: Lamin A; yellow: LaminC). Ponceau staining was used as a loading control. **n.** *Upper panel*: representative AFM line profiles showing nuclear height (solid lines) and indentation depth (dotted lines) across neuronal nuclei from wild-type (blue) and MeCP2 knockout (red) conditions. Vertical dashed lines indicate the lateral nuclear boundaries. Although the maximum indentation could exceed 1.5 μm in some regions, most indentation values remained well within the 1 μm fitting range (yellow area). These profiles illustrate the relationship between nuclear height and local deformation under applied load, supporting the choice of a 1 μm indentation limit for elastic-modulus fitting. *Lower panel*: Representative force–indentation (F–δ) curves extracted from the positions marked by asterisks in the upper panel. Curves followed a power law $F \propto \delta^m$ with $m \approx 2$, as fitted using Igor Pro 6.37 (WaveMetrics, Inc., Portland, OR), consistent with elastic conical contact, with fitted regions shown as blue traces over the experimental curves. The shaded yellow region indicates the indentation range (≤ 1 μm) used for contact-mechanics fitting, which remained within the elastic regime. At higher indentation, fitting $F=C\delta^2$ yielded C=270.9±4.5 with minimal residual deviation between data and fit ($\chi^2 = 1.3\times10^{-18}$); using the Sneddon cone relation (θ=25°, ν=0.5), the corresponding Young's modulus was E=0.68±0.01 kPa. At lower indentation, C=36,555±683 ($\chi^2 = 5.8\times10^{-18}$) gave E=92.4±1.7 kPa (θ=25°, ν=0.5). In both cases, the fits follow the expected elastic power law $F \propto \delta^2$.

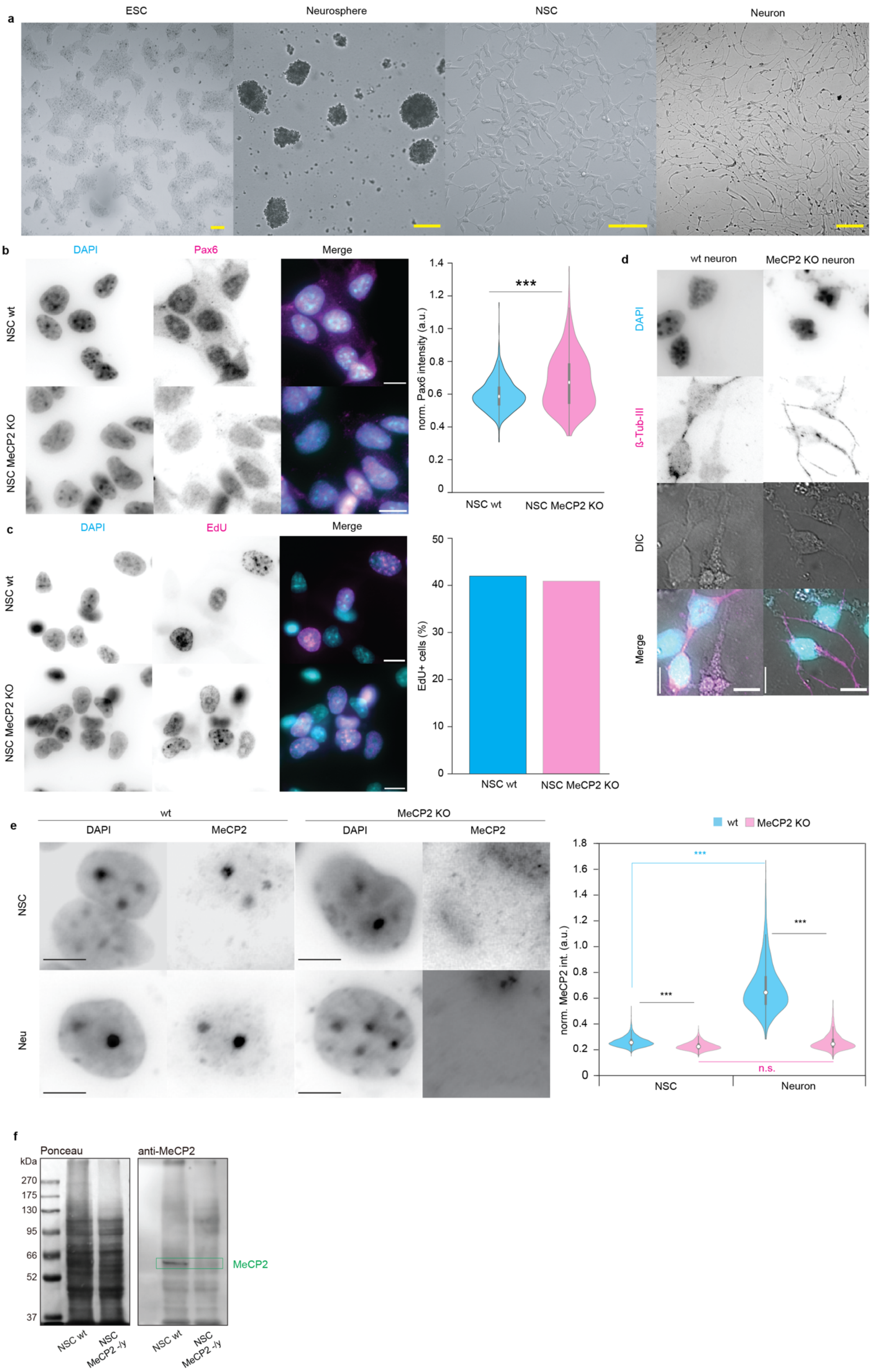

a
ESC
Neurosphere
NSC
Neuron
b
DAPI
Pax6
Merge
NSC wt
NSC MeCP2 KO
norm. Pax6 intensity (a.u.)
***
NSC wt
NSC MeCP2 KO
c
DAPI
EdU
Merge
NSC wt
NSC MeCP2 KO
EdU+ cells (%)
NSC wt
NSC MeCP2 KO
d
wt neuron
MeCP2 KO neuron
DAPI
ß-Tub-III
DIC
Merge
e
wt
MeCP2 KO
DAPI
MeCP2
DAPI
MeCP2
NSC
Neu
wt
MeCP2 KO
norm. MeCP2 int. (a.u.)
***
***
***
n.s.
NSC
Neuron
f
Ponceau
anti-MeCP2
kDa
270
175
130
95
66
52
37
MeCP2
NSC wt
NSC MeCP2 -/y
NSC wt
NSC MeCP2 -/y

Supplementary Figure 4. Generation and characterization of J1 NSC wt and J1 NSC MeCP2 KO.

a. Overview images of the steps of the generation of NSC from Figure 3d and its differentiation to neurons. Scale bar: 100 µm. **b.** Immunostaining of the neural stem cell marker Pax6 normalized to the DAPI (DNA) intensity. Representative images and quantification of Pax6 intensity with high throughput microscopy. Scale bar: 10 µm. **c.** Visualization of the replication potential of the NSC by EdU incorporation and quantification of EdU+ cells. >500 cells from 3 biological replicates were counted for each condition. Scale bar: 10 µm. **d.** Representative images of immunostaining of wt and MeCP2 KO cells after 21 days of differentiation with the neuronal marker ß-Tubulin-III. Scale bar: 10 µm. **e.** Representative images of the immunostaining of MeCP2 (scale bar: 10 µm) and quantification of MeCP2 in wt and MeCP2 KO NSCs and neurons (Neu). Quantification of MeCP2 intensity was normalized to DAPI intensity due to the bigger area of MeCP2 KO neurons compared to wt. In the violin plots, the x spread represents the frequency of the data in the corresponding y, the gray box represents the 1st and 3rd percentiles, the white dot the median, and the whiskers the standard deviation. Significance was defined based on the p-values obtained from a two-sided t-test. n.s.: non-significant (p-value > 0.05) *: p-value < 0.05; **: p-value < 0.001; ***: p-value < 0.0001. **f.** Western blot of MeCP2 in NSC wt and NSC MeCP2 KO cells to confirm the absence of the protein in the MeCP2 KO cells. MeCP2 expected size is marked with a green square. Ponceau staining was used as a loading control.

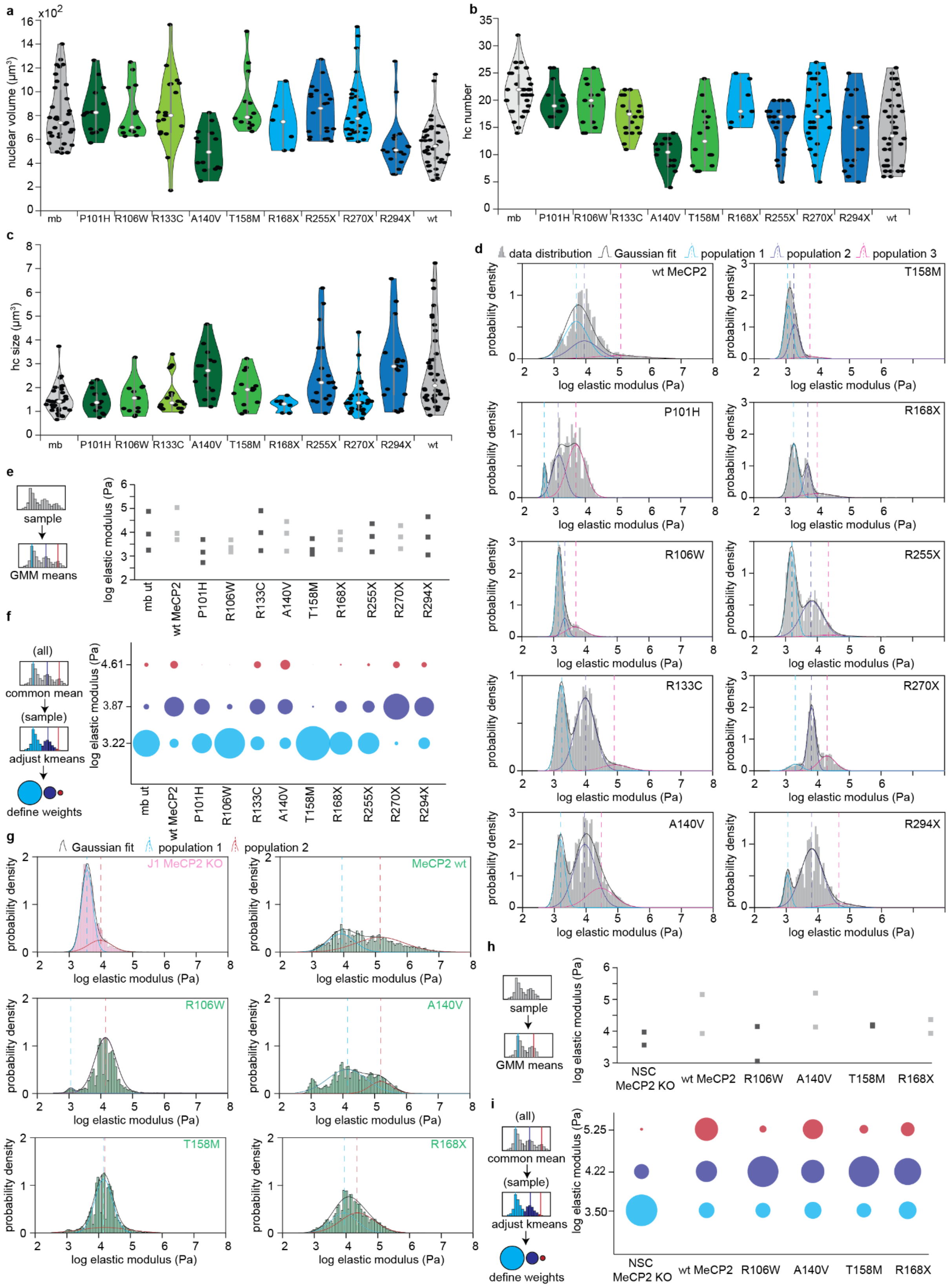

Supplementary Figure 5. Supplementary information for figure 4.

a-c. 3D confocal analysis for C2C12 cells transfected with MeCP2 wt and Rett mutations. The untransfected sample from Figure 2 was added for comparison. Cells were fixed in formaldehyde and DNA was stained with DAPI, and used to segment and quantify the nuclear volume (a), as well as the

heterochromatin compartment number (b) and average size (c). In the violin plots, the x spread represents the frequency of the data in the corresponding y, the gray box represents the 1st and 3rd percentiles, the white dot the median, and the whiskers the standard deviation. **d-f.** Elastic modulus analysis for C2C12 transfected with MeCP2 wt or Rett mutations. Histograms of the elastic modulus values (d) were fitted to a 3 populations Gaussian mixture model (black lines: full model; colored lines: individual populations), and the means of these populations were compared (e). Additionally, all data were plotted together to extract common populations, and the individual samples were adjusted to these populations to quantify the weight of each population (f) using k-means. The results of e and f were used as the input to generate the dendrogram shown in Figure 4d. **g-i.** Elastic modulus analysis for NSC MeCP2 KO rescue with MeCP2 wt and Rett mutations. As described before, the histogram of the elastic modulus values (g) was fitted to a 2 population Gaussian mixture model and the mean were compared (h). Analysis of common populations weights was done using k-means (i). The results shown in h and g were used to perform the dendrogram shown in Figure 4f.

Supplementary Table 1. Cell lines used and their characteristics.

Name	Species	Type	Genotype	Reference
C2C12	*Mus musculus*	myoblast	wild type	[113]
J1 wt	*Mus musculus*	embryonic stem cell	wild type	[114]
J1 MIN-MeCP2	*Mus musculus*	embryonic stem cell	wild type	this study
J1 MeCP2 KO	*Mus musculus*	embryonic stem cell	wild type	this study
J1 NSC wt	*Mus musculus*	neural stem cell	wild type	this study
J1 NSC MeCP2 KO	*Mus musculus*	neural stem cell	wild type	this study

Supplementary Table 2. Oligos used in this work.

Name	Sequence (5´-3´)	Use	Reference
MeCP2-transcript2N-gRNA-F	CACCGCAGCTACCATGGAATCCTGT	CRISPR/ Cas9	this study
MeCP2-transcript2N-gRNA-R	AAACACAGGATTCCATGGTAGCTGC	CRISPR/ Cas9	this study
MeCP2ex2-FW (F)	GCACTCAAGCTCACCTATACTC	screening	this study
MeCP2ex2-RV (R)	CTGAAGGTTGTAGTGGCTCAT	screening	this study
attL-FW (F2)	GACAGGAACTCCTGCCAAT	screening	this study
pEG-hMECP2-BB-F-2	TGACTTTACACGGAGGATCCACCG	cloning	[35]
hMeCP2-270X-RV	GCCCCGTTTCTTGGGAAT	cloning	this study
hMeCP2-294X-RV	GATAGAAGACTCCTTCACG	cloning	this study
mNotch1 fw	CCCTTGCTCTGCCTAACGC	qPCR	[115]
mNotch1 rev	GGAGTCCTGGCATCGTTGG	qPCR	[115]
mNotch2 fw	GCAGGAGCAGGAGGTGATAG	qPCR	[115]
mNotch2 rev	GCGTTTCTTGGACTCTCCAG	qPCR	[115]
Tgfbr1 fw	ATCACCTGGCCTTGGTCCTGTGG	qPCR	[116]
Tgfbr1 rv	GGTCCTCTTCATTTGGCACTCGATG	qPCR	[116]
Gadph fw	CCAACATACAGGTTTCTCCAG	qPCR	[39]
Gadph rv	CTGGAAAGCTGTGGCGTGATGG	qPCR	[39]

Supplementary Table 3. Plasmid used in this work.

Name	Pc[a] number	Fluorophore	Gene species	Promoter	Addgene	Reference
pEG-MeCP2	1208	EGFP	*Homo sapiens*	CMV		[117]
pEG-MeCP2_P101H	1246	EGFP	*Homo sapiens*	CMV		[117]
pEG-MeCP2_R106W	1266	EGFP	*Homo sapiens*	CMV		[117]
pEG-MeCP2_R133C	1254	EGFP	*Homo sapiens*	CMV		[117]
pEG-MeCP2_A140V	1261	EGFP	*Homo sapiens*	CMV		[117]
pEG-MeCP2_T158M	1265	EGFP	*Homo sapiens*	CMV		[117]
pEG-MeCP2_R168X	4748	EGFP	*Homo sapiens*	CMV	248578	[35]
pEG-MeCP2_R255X	4749	EGFP	*Homo sapiens*	CMV	248577	[35]
pEG-MeCP2_R270X	4751	EGFP	*Homo sapiens*	CMV	248579	this study
pEG-MeCP2_R294X	4753	EGFP	*Homo sapiens*	CMV	248580	this study
pSpCas9(BB)-2A-Puro (PX459) V2.0	3926	-	*Streptococcus pyogenes*	Cbh	62988	[118]
pCAG-NLS-Bxb1	5126	-	Mycobacteriophage	CAG	65625	[100]
pattB-Cherry-stop-puro	5127	mCherry	*Discosoma* sp.	-	65529	[100]

[a] pc: plasmid collection

Supplementary Table 4. Antibodies used in this work.

Reactivity	Host	Fixation (IF[a])	Dilution	Application	Catalog / Clone	Company / Reference
anti-LaminAC	rabbit	methanol	1:200/ 1:1000	IF[a] / WB[b]	-	Provided by Dr. Brian Burke
anti-Lamin B	mouse	methanol	undiluted/ 1:10	IF[a] / WB[b]	X223	Progen Biotechnik (Germany)
anti-MeCP2	mouse	formaldehyde	undiluted	IF[a]	4B4	[119]
anti-MeCP2	rat	formaldehyde / methanol	undiluted	IF[a] / WB[b]	4H7	[119]
anti-NeuN	rabbit	formaldehyde	1:500	IF[a]	EPR12763	Abcam (United Kingdom)
anti-Oct3/4	mouse	formaldehyde	1:500	IF[a]	Clone 50/ Oct-3 (RUO)	BD Biosciences (Germany)
anti-Pax6	rabbit	formaldehyde	1:100	IF[a]	901301	Biolegend (United Kingdom)
Anti-Tubulin ß-III	mouse	formaldehyde	1:60	IF[a]	MAB 1195	Bio-Techne GmbH (Germany)
anti-mouse IgG Alexa Fluor 488	goat	formaldehyde / methanol	1:800	IF[a]	2120125	Invitrogen (Germany)
anti-mouse IgG Cy5	donkey	formaldehyde / methanol	1:400	IF[a]	711-175-150	Jackson Immuno Research (United Kingdom)
anti-rabbit IgG Alexa Fluor 647	goat	formaldehyde / methanol	1:400	IF[a]	2160406	Molecular Probes (Germany)
anti-rabbit IgG Cy5	donkey	formaldehyde / methanol	1:500	IF[a]	711-175-152	Jackson Immuno Research (United Kingdom)
anti-rat IgG (H+L) HRP[c]-conjugated	goat	-	1:1000	WB[b]	A9037	Sigma-Aldrich (Germany)
anti-rat IgG Alexa Fluor 488	donkey	formaldehyde / methanol	1:800	IF[a]	A_11006	Invitrogen (Germany)

[a] Immunofluorescence; [b] Western blot; [c] horseradish peroxidase

Supplementary Table 5. Microscopy and cell sorting devices and their characteristics.

Microscope / Company	Laser / Lamps	Filters (ex[a] & em[b] [nm])	Objective / Lenses	Detection system	Application
S3e Cell Sorter / BioRad laboratories	488 nm	GFP (em: 525±30)		FSC[c] with PMT[d] SSC[e] with PMT[d] Fluorescence detectors with PMTs[d]	Sort cells according to GFP expression
Axio Observer Z1 optical microscope /Carl Zeiss	HAL 100	–	20x air LD A-Plan 20x/0.35 M27 (FWD=4.9mm at CG=1mm polystyrene)	Axiocam 506 color 6 MPx 16mm Chip size	AFM optical visualization
Nikon Eclipse TiE2 / Nikon	Spectra X Led 395±25 (295 mW) 470±24 nm (196 mW) 540±30 nm (231 mW)	em.: Quadbandpass (432±25, 515±25, 595±25, 730±70 nm)	20X air Splan Fluor LWD DIC (0.7 Na[f], 2.3 mm WD[g]) 40X air Plan Apo l DIC (0.95 Na[f], 230 µm WD[g])	Nikon Qi2 751600 16.25 MPx	Widefield High throughput microscopy
Leica TSC SPE-II	Multicolor solid-state laser module RYBC 405 nm (25mW) 488 nm (10 mW) 635 nm (18mW)	em.: DAPI: 410-600 GFP: 493-540 Cy5: 640-783	ACS APO 63X (1.3 NA[f], 0.16 WD[g])	Trans-PMT[d]	3D confocal

[a] ex: excitation; [b] em: emission;[c] FSC: forward scatter; [d] PTM: photomultiplier tube(s); [e] SSC: side scatter;[f] NA: numerical aperture; [g] WD: working distance

Supplementary Table 6. Statistics of confocal 3D analyses of fixed cells or purified (not fixed) nuclei counterstained with DAPI. Values are given as mean ± 95% confidence interval. * This individual condition was measured using AFM rather than 3D confocal analysis.

**Condition**	**n**	**hc number**	**average hc size (μm^3)**	**nuclear volume (μm^3)**	**HOI**
C2C12 mb	35	22.9 ± 1.5	1.487 ± 0.205	803.87 ± 88.94	8.51x10^{-5}
C2C12 nuclei purified	81	–	–	859.78 ± 47.78	–
C2C12 nuclei seeded	35	–	–	814.51 ± 94.28	–
C2C12 nuclei in AFM*	7	–	–	787.70 ± 175.92	–
C2C12 +pMeCP2 low	13	15.6 ± 3.1	1.702 ± 0.454	638.43 ± 126.36	17.07x10^{-5}
C2C12 +pMeCP2 high	10	7.0 ± 1.4	3.822 ± 1.339	402.43 ± 87.53	135.69x10^{-5}
C2C12 +pMeCP2	40	14.4 ± 6.0	1.653 ± 0.529	559.85 ± 189.27	33.80x10^{-5}
C2C12 +pMeCP2 P101H	15	19.3 ± 2.4	1.439 ±0.267	845.57 ± 107.62	8.54x10^{-5}
C2C12 +pMeCP2 R106W	13	19.3 ± 2.4	1.535 ± 0.416	832.47 ± 222.23	9.55x10^{-5}
C2C12 +pMeCP2 R133C	20	17.3 ± 1.6	1.649 ± 0.344	839.08 ± 139.43	11.40x10^{-5}
C2C12 +pMeCP2 A140V	14	10.1 ± 1.6	2.977 ± 1.200	511.77 ± 103.21	57.30x10^{-5}
C2C12 +pMeCP2 T158M	14	13.3 ± 3.1	1.846 ± 0.414	891.30 ± 142.01	15.60x10^{-5}
C2C12 +pMeCP2 R168X	7	19.4 ± 3.7	1.262 ± 0.252	730.83 ± 194.02	8.89x10^{-5}
C2C12 +pMeCP2 R255X	20	15.3 ± 2.1	2.589 ± 0.689	834.47 ± 96.96	20.30x10^{-5}
C2C12 +pMeCP2 R270X	29	17.7 ± 2.2	1.603 ± 0.302	850.40 ± 98.76	10.60x10^{-5}
C2C12 +pMeCP2 R294X	19	13.7 ± 3.0	2.847 ± 0.754	559.03 ± 109.49	37.20x10^{-5}
J1 wt -LIF D7	39	19.0 ±1.8	1.247 ± 0.147	1312.42 ± 121.85	5.00x10^{-5}
J1 wt -LIF D14	16	14.2 ± 4.4	1.950 ± 0.687	1272.45 ± 219.80	10.79x10^{-5}
J1 wt -LIF D21	38	8.7 ± 1.5	2.766 ± 0.802	855.77 ± 144.08	37.33x10^{-5}
J1 MeCP2 KO -LIF D7	28	17.1 ± 1.6	1.065 ± 0.215	1049.74 ± 96.75	5.93x10^{-5}
J1 MeCP2 KO -LIF D14	32	17.1 ± 1.9	1.042 ± 0.162	1229.09 ± 216.58	4.95x10^{-5}
J1 MeCP2 KO -LIF D21	12	16.1 ± 2.4	1.010 ± 0.509	1405.03 ± 645.94	4.47x10^{-5}
J1 wt NSC	85	12.7 ± 0.8	1.871 ± 0.149	146.96 ± 7.74	100.21x10^{-5}
J1 wt neuron	61	4.7 ± 0.6	3.663 ± 0.708	144.74 ± 6.74	534.11x10^{-5}
J1 MeCP2 KO NSC	87	13.8 ± 0.9	1.692 ± 0.132	161.07 ± 8.46	76.14x10^{-5}
J1 MeCP2 KO neuron	48	15.0 ± 1.4	2.107 ± 0.194	193.16 ± 20.23	72.53x10^{-5}

hc: heterochromatin compartment; HOI: heterochromatin organization index.

Supplementary Table 7. Statistics of intensity measurements. Values of intensity are given as mean ± 95% confidence interval.

condition	n	method	factor	intensity (a.u.)
C2C12 myoblast	22	confocal	Lamin B^m	12.878 ± 2.364
C2C12 +pMeCP2 low	10	confocal	LaminB^m	11.677 ± 3.478
C2C12 +pMeCP2 high	13	confocal	LaminB^m	12.563 ± 4.256
C2C12 myoblast	22	confocal	LaminAC^m	16.130 ± 3.062
C2C12 +pMeCP2 low	10	confocal	LaminAC^m	6.595 ± 1.845
C2C12 +pMeCP2 high	13	confocal	LaminAC^m	7.546 ± 2.183
J1 wt ESC	196	high-throughput	MeCP2^n	0.280 ± 0.008
J1 wt -LIF D7	160	high-throughput	MeCP2^n	0.316 ± 0.010
J1 wt -LIF D14	136	high-throughput	MeCP2^n	0.435 ± 0.012
J1 wt -LIF D21	117	high-throughput	MeCP2^n	0.684 ± 0.040
J1 MeCP2 KO -LIF D7	63	high-throughput	MeCP2^n	0.206 ± 0.013
J1 MeCP2 KO -LIF D14	16	high-throughput	MeCP2^n	0.191 ± 0.014
J1 MeCP2 KO -LIF D21	74	high-throughput	MeCP2^n	0.272 ± 0.019
J1 wt -LIF D7	153	high-throughput	NeuN^n	0.438 ± 0.027
J1 wt -LIF D14	102	high-throughput	NeuN^n	0.828 ± 0.065
J1 wt -LIF D21	182	high-throughput	NeuN^n	1.025 ± 0.048
J1 MeCP2 KO -LIF D7	60	high-throughput	NeuN^n	0.442 ± 0.017
J1 MeCP2 KO -LIF D14	205	high-throughput	NeuN^n	0.866 ± 0.028
J1 MeCP2 KO -LIF D21	135	high-throughput	NeuN^n	1.043 ± 0.043
J1 wt ESC	258	high-throughput	Oct4^m	6452.2 ± 215.4
J1 wt -LIF D7	152	high-throughput	Oct4^m	3503.6 ± 86.4
J1 wt -LIF D14	191	high-throughput	Oct4^m	2176.9 ± 33.0
J1 MeCP2 KO ESC	79	high-throughput	Oct4^m	6244.7 ± 320.7
J1 MeCP2 KO -LIF D7	110	high-throughput	Oct4^m	3626 ± 755.8
J1 MeCP2 KO -LIF D14	6	high-throughput	Oct4^m	2581.2 ± 424.9
J1wt NSC	3500	high-throughput	LaminAC	$3.52x10^5 \pm 7.70x10^3$
J1 MeCP2 KO NSC	3500	high-throughput	LaminAC	$3.28x10^5 \pm 6.97x10^3$
J1 wt NSC	5207	high-throughput	LaminB	$3.23x10^5 \pm 5.36x10^3$
J1 MeCP2 KO NSC	5207	high-throughput	LaminB	$3.40x10^5 \pm 5.69x10^3$
J1wt NSC	18754	high-throughput	Pax6	$2.77x10^5 \pm 1.53x10^3$
J1 MeCP2 KO NSC	45280	high-throughput	Pax6	$1.83x10^5 \pm 9.43x10^2$
J1wt NSC	17424	high-throughput	MeCP2^n	0.337 ± 0.001
J1 MeCP2 KO NSC	16258	high-throughput	MeCP2^n	0.290 ± 0.001
J1 wt neuron	8935	high-throughput	MeCP2^n	0.703 ± 0.004
J1 MeCP2 KO neuron	4150	high-throughput	MeCP2^n	0.336 ± 0.003

^{m}: mean intensity; ^{n}: normalized to DAPI intensity

References for Supplementary information

113. Yaffe, D., and Saxel, O. (1977). Serial passaging and differentiation of myogenic cells isolated from dystrophic mouse muscle. Nature *270*, 725–727. 10.1038/270725a0.

114. Li, E., Bestor, T.H., and Jaenisch, R. (1992). Targeted mutation of the DNA methyltransferase gene results in embryonic lethality. Cell *69*, 915–926. 10.1016/0092-8674(92)90611-f.

115. Trujillo-Paredes, N., Valencia, C., Guerrero-Flores, G., Arzate, D.-M., Baizabal, J.-M., Guerra-Crespo, M., Fuentes-Hernández, A., Zea-Armenta, I., and Covarrubias, L. (2016). Regulation of differentiation flux by Notch signalling influences the number of dopaminergic neurons in the adult brain. Biol. Open *5*, 336–347. 10.1242/bio.013383.

116. Pohlers, D., Beyer, A., Koczan, D., Wilhelm, T., Thiesen, H.-J., and Kinne, R.W. (2007). Constitutive upregulation of the transforming growth factor-beta pathway in rheumatoid arthritis synovial fibroblasts. Arthritis Res. Ther. *9*, R59. 10.1186/ar2217.

117. Kudo, S., Nomura, Y., Segawa, M., Fujita, N., Nakao, M., Schanen, C., and Tamura, M. (2003). Heterogeneity in residual function of MeCP2 carrying missense mutations in the methyl CpG binding domain. J. Med. Genet. *40*, 487–493. 10.1136/jmg.40.7.487.

118. Ran, F.A., Hsu, P.D., Wright, J., Agarwala, V., Scott, D.A., and Zhang, F. (2013). Genome engineering using the CRISPR-Cas9 system. Nat. Protoc. *8*, 2281–2308. 10.1038/nprot.2013.143.

119. Jost, K.L., Rottach, A., Milden, M., Bertulat, B., Becker, A., Wolf, P., Sandoval, J., Petazzi, P., Huertas, D., Esteller, M., et al. (2011). Generation and characterization of rat and mouse monoclonal antibodies specific for MeCP2 and their use in X-inactivation studies. PLoS ONE *6*, e26499. 10.1371/journal.pone.0026499.
